# Learning in public goods games: the effects of uncertainty and communication on cooperation

**DOI:** 10.1007/s00521-024-10530-6

**Published:** 2025-01-30

**Authors:** Nicole Orzan, Erman Acar, Davide Grossi, Roxana Rădulescu

**Affiliations:** 1 Bernoulli Institute for Mathematics, Computer Science and Artificial Intelligence, University of Groningen, 9747 AG Nijenborgh, Groningen, The Netherlands; 2 Institute for Logic, Language and Computation, University of Amsterdam, 1098XH Science Park, Amsterdam, The Netherlands; 3 Informatics Institute, University of Amsterdam, 1098XH Science Park, Amsterdam, The Netherlands; 4 Amsterdam Center for Law and Economics, University of Amsterdam, Nieuwe Achtergracht 166, 1018 WV Amsterdam, The Netherlands; 5 Intelligent Systems Group, Utrecht University, Princetonplein 5, 3584 CC Utrecht, The Netherlands; 6 Artificial Intelligence Lab, Vrije Universiteit Brussel, Pleinlaan 2, 1050 Brussels, Belgium

**Keywords:** Multi-agent reinforcement learning, Emergent communication, Social dilemmas

## Abstract

Communication is a widely used mechanism to promote cooperation in multi-agent systems. In the field of emergent communication, agents are typically trained in specific environments: cooperative, competitive or mixed-motive. Motivated by the idea that real-world settings are characterized by incomplete information and that humans face daily interactions under a wide spectrum of incentives, we aim to explore the role of emergent communication when simultaneously exploited across all these contexts. In this work, we pursue this line of research by focusing on social dilemmas. To do this, we developed an extended version of the Public Goods Game, which allows us to train independent reinforcement learning agents simultaneously in different scenarios where incentives are (mis)aligned to various extents. Additionally, agents experience uncertainty in terms of the alignment of their incentives with those of others. We equip agents with the ability to learn a communication policy and study the impact of emergent communication in the face of uncertainty among agents. Our findings show that in settings where all agents have the same level of uncertainty, communication can enhance the cooperation of the whole group. However, in cases of asymmetric uncertainty, the agents that do not face uncertainty learn to use communication to deceive and exploit their uncertain peers.

## Introduction

Cooperation is a fundamental feature of human societies. Traditionally, its emergence among self-interested agents has been considered a challenge by disciplines concerned with human interaction, such as biology or sociology [51]. More recently, cooperation has also been gaining a central stage in artificial intelligence research: the ability to cooperate is now considered an essential feature for artificial agents to operate meaningfully within human societies [1, 13]. A substantial body of the literature already exists on the emergence of cooperation among artificial agents [12, 48, 49]. A big chunk of the literature in this field focuses on agents learning to cooperate in cooperative environments, where incentives are aligned and cooperation is therefore clearly beneficial [24, 42, 62]. However, more recently researchers have begun to address the challenge of the emergence of cooperation in more realistic and complex settings. These often involve environments where the incentives for cooperation are weaker, because agents’ incentives are less clearly aligned [3, 8, 9, 46]. These scenarios are referred to as mixed-motives environments, a term first introduced by Schelling in 1958 [52]. Typical instances of such environments are those involving interactions known in economics and sociology as social dilemmas [14, 33], which model situations where individual goals conflict with collective welfare. The present paper focuses on one such social dilemma, known as the “public goods game” [2], taking a reinforcement learning (RL) [55] perspective.

Within the multi-agent reinforcement learning (MARL) framework, agents are usually trained to act optimally in either cooperative, competitive or mixed-motives environments [7, 63]. However, whether and how cooperation emerges in humans and animals depends on interactions that occur across environments with varying degrees of incentives’ (mis-)alignment. This phenomenon is supported by a number of studies in the field of social psychology, which show how humans are able to elaborate effective cooperative or competitive strategies depending on changes in their environment [17, 18, 31]. Inspired by the acknowledgement that human real-world contexts consist of interactions under a various spectrum of incentives, we believe it is important to investigate the learning outcomes of RL agents when trained in a spectrum of environments with incentives that are (mis)aligned to varying degrees. We argue this to be essential for the development of MARL-based applications capable of handling the complexity of real-world scenarios.

Apart from the coexistence of cooperative and selfish goals, another element that significantly challenges the emergence of cooperation among humans, as well as artificial agents, is uncertainty. Uncertainty can present itself in two main forms: social uncertainty is related to the lack of information about the opponent’s moves history and expected behaviour [4, 16], while environmental uncertainty refers to the lack of knowledge on the game’s payoff structure, including the preferences or incentives of other agents [3, 61]. As a result, environmental uncertainty can leave agents unsure about whether they are operating in a cooperative, competitive or mixed-motive environment. In this work, we refer to such type of environmental uncertainty as *incentive uncertainty*.

To the best of our knowledge, no prior research has investigated the behaviour of RL agents trained across a spectrum of environments with varying degrees of incentive alignment and with environmental uncertainty regarding such (mis-)alignment. This study has the primary objective of examining the impact of incentive uncertainty on cooperation and assessing whether emergent communication, characterized by “cheap talk” (i.e. non-binding and costless signals), can help enhance it. We present the following key *contributions*:

- We develop a multi-agent environment based on the Public Goods Game, which we refer to as the Extended Public Goods Game (EPGG). This environment allows training agents on a spectrum of games ranging from fully cooperative (i.e. the cooperative strategy is dominant and optimal), to mixed-motives (i.e. the non-cooperative/defective strategy is dominant but suboptimal), to fully competitive (i.e. the non-cooperative/defective strategy is dominant and optimal).
- Within the EPGG, we analyse how incentive uncertainty influences the cooperative behaviour of independent RL agents. We find that when incentive uncertainty is introduced during the training of agents across a variety of environments with differing levels of incentives’ alignment, it results in a reduction of cooperative behaviour among agents in cooperative and mixed-motive games.
- We explore the influence of emergent communication in the EPGG. We empirically show that in situations where all the agents experience uncertainty, the introduction of a communication channel between agents can help mitigate the negative effects of uncertainty, specifically shifting the learning outcome towards cooperation. Moreover, when uncertainty is asymmetric, certain agents may learn to employ communication to deceive others.
- We furthermore observe that the addition of communication in the case of environmental uncertainty allows agents to identify beneficial cooperation more effectively than in the case in which agents have perfect knowledge of the environment. This result offers an intriguing route to support cooperation in situations where incentives are not completely aligned.

The rest of the paper is structured as follows: in Sect. 2, we provide an overview of related work, focusing on emergent communication via reinforcement learning and social dilemmas. We then introduce the necessary preliminaries (Sect. 3), specifically the Public Goods Game and the Extended Public Goods Game. In Sect. 4, we describe the methods employed in this work, namely policy-based reinforcement learning algorithms, and we describe the scenarios with and without communication, the implementation of uncertainty and the metrics we employ to monitor the emergent communication. In Sect. 5, we present findings for both the two-player and three-player scenarios, focusing on the average cooperation levels achieved by the agents, emergent communication metrics and statistical analysis of the results. Finally, we conclude by summarizing the outcomes and discussing avenues for future work (Sect. 6).

## Related work

Our work builds on previous research in the fields of emergent communication and social dilemmas within multi-agent reinforcement learning.

*Emergent communication.* Seminal works in the emergent communication field were published by Foerster et al. [23, 25], which studied a sequential decision-making problem in a partially observable multi-agent system. Later, [54] used a continuous communication protocol to solve cooperative tasks, and [53] was the first to work on mixed and competitive tasks. In subsequent years the field of emergent communication took off—with an overview presented in [32, 35]—and many papers focused on solving referential games, where a pair of sender and receiver agents have to learn a communication protocol to solve cooperative tasks [21, 26, 36, 37].

Most of the work concerning the emergence of communication focused on cooperative scenarios. Nonetheless, the study of mixed scenarios remains highly relevant, since real-world scenarios frequently encompass elements of competition and self-interest, which have been relatively less explored in the field of emergent communication. One of the papers focusing on this setting is the one by Cao et al. [8], studying a bargaining negotiating scenario in which agents use emergent communication to split a common pool of items. This work explores how communication aids prosocial agents but neglects the self-interested case. In addition to competition among individual agents, some studies have explored the role of emergent communication among competing teams of agents. An example is the work of Liang et al. [41], where the authors analyse emergent communication in the game “Task, Talk & Compete”. Similar work has been developed by Vanneste et al. [58], where the focus is the prey–predator game, with the addition of an (emergent) communication channel among predators; as well as by Brandizzi et al. [6], where the authors explore the role of emergent communication in the social deduction Werewolf game, as a tool to improve the performance of the playing teams. In this landscape, [46] tackled a mixed cooperative–competitive signalling game. Their setting defines a non-situated game^1^ with a variable amount of cooperation. These characteristics make this setting similar to ours; our unique distinction lies in our simultaneous learning across various environments, each featuring a different incentive alignment level, and our incorporation of uncertainty. Our scenario aims to reflect better the complexity of the real world, which presents scenarios with variable and uncertain incentives. Moreover, we tackle social dilemmas that are scalable to any number of agents, as opposed to typical referential tasks that often involve only two agents. Crucially, these features distinguish our study from the previously mentioned works, steering our research towards understanding emergent communication in complex, real-world scenarios.

*Social dilemmas.* As Kollocks defines them in 1998, “Social Dilemmas are situations where individual rationality leads to collective irrationality” [33], and are characterized by the presence of suboptimal equilibria, namely outcomes which are less favourable than others for all the members of the group. Social dilemmas have been studied in game theory, psychology and economics. Researchers have focused on how to solve social dilemmas avoiding suboptimal competitive equilibria and increase cooperation among the agents. A common outcome of these studies is the positive effect of communication on the cooperation level of the agents [15, 33, 34]. Communication among players has also been studied from a game-theoretic perspective: in [10], the authors focused on information exchange in a sender–receiver signalling game and concluded that perfect communication occurs only when agents’ goals are perfectly aligned, and the more the incentives of the agents are misaligned, the more it declines.

In addition to communication, intrinsic motivation has been studied as a tool that helps foster cooperation in mixed-motive scenarios [29]. This concept characterizes the drive to do something for its inherent enjoyability rather than for any external or utilitarian gain, defining a reward signal that does not come from external sources but rather from the agent itself.

However, what happens when players are uncertain about the payoff they will receive from the game? In the context of the public goods game, the real-world production of public goods may depend on a partially known function (similar to estimating the income of a newly produced movie): this number could be estimated, but not known beforehand. The effect of uncertainty with respect to the payoffs in the public goods game has been studied extensively in the literature [5, 19, 22, 39]; different works show that, when players are uncertain about the payoff, their contributions to the public good significantly decrease.

Our study adopts a reinforcement learning perspective to investigate emergent cooperation in social dilemmas. The previous literature in this field has used RL to explore sequential social dilemmas, which are temporally extended games with game-theoretic payoff matrices. In this landscape, [11] and [38] focus on agents learning cooperation in multi-agent social dilemmas without the use of communication. O’Callaghan et al. [47] use multi-objective reinforcement learning to tune the cooperative–competitive behaviour of agents. In [28], the authors implement fairness norms to solve dilemmas, and in [29], influence rewards are used to enhance coordination and communication among agents. Our unique contribution sets us apart from this body of work as we combine elements of incentive uncertainty, emergent communication and reinforcement learning. We explore this in the framework of the public goods game, which has been previously studied with a reinforcement learning perspective only in [44], where the authors implemented a simulator an RL-based simulator of agents playing the game. Our approach builds upon studies that utilize reinforcement learning to address social dilemmas and mixed-motive games. What distinguishes our approach is the incorporation of training across various games with different levels of incentive alignment and uncertainty regarding this alignment.

## Preliminaries

In this section, we define the Public Goods Game and discuss its equilibria, and introduce our proposed extension.

### The public goods game

**Fig. 1:**
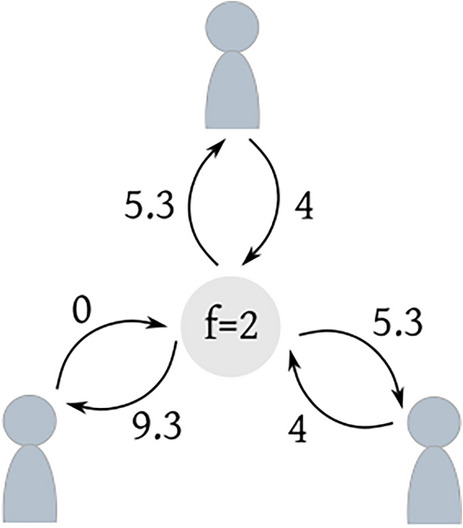
Representation of the public goods game with three players and multiplication factor $f=2$

A public good is an asset that provides benefit to all individuals in a social group, both the ones that participated in its production and the ones who did not [33]. For a selfish agent, whose primary goal is to maximize its own utility, the rational strategy when confronted with the choice to contribute to the public good or not, is to free ride: profit from the public good without investing resources [2]. This strategy is not only driven by the goal of maximizing individual utility but also reinforced by the fear that others may not cooperate in the production of the public good, leading the individual to lose their investment [33]. However, if every individual follows the purely selfish strategy, the public resource would not be produced, and no one would benefit from it. In this game, the players’ incentives are neither perfectly aligned nor misaligned. Using the terminology of [52], we refer to this type of interaction where the utilities of the players are affected by the presence of partial conflict and interdependence, as “mixed-motive” (Fig. 1).

***Formal definition of public good games***

We start by defining the public goods games formally.

#### Definition 1

(*Public Goods Game*) A Public Goods Game (PGG) is a tuple $\langle N, \varvec{c}, A, f, \varvec{u} \rangle$, where

- *N* is the set of players, and $|N| = n \in \mathbb {N}$ is the number of players, with $n>1$.
- $\varvec{c} = (c_1, \ldots , c_n)$ with $c_i \in \mathbb {R}$, for each $i \in N$ is the tuple of endowments, that is, the number of coins or wealth that each agent possesses;
- $A = \{C, D\}$ is the set of actions available to the agents, namely every player can either cooperate (*C*) investing the full endowment in the public good or defect (*D*) with no investment. We furthermore denote by $\varvec{a} = (a_1, \dots , a_n) \in A^n$ the action profiles, that is, the tuple containing the actions chosen by each agent.
- $f \in \mathbb {R}_{\ge 0}$ is the multiplication factor, that is, the factor by which the collective investment is multiplied to generate the public good.
- $\varvec{u}$ is the tuple of agents’ utilities. The utility for agent *i* determines the number of coins (or goods) the agent receives, given the action profile $\varvec{a}$, the multiplicative factor *f* and the agents’ endowments $\varvec{c}$. Therefore, the utility for agent *i* is a function $u_i: A^{n} \times \mathbb {R}_{\ge 0} \times \mathbb {R}^n \rightarrow \mathbb {R}$, defined as:
  1 $$\begin{aligned} u_i(\varvec{a}, f, \varvec{c}) = \frac{1}{n} \sum _{j=1}^{n} c_j I(a_j) \cdot f + c_i (1 - I(a_i)), \end{aligned}$$
  where $a_j$ denotes the *j*th entry of ${\varvec{a}}$ and $I(a_j)$ is the indicator function:
  2 $$\begin{aligned} I(a_j) = {\left\{ \begin{array}{ll} 1 &  \quad {\text {if }} a_j = C\\ 0 &  \quad {\text {otherwise.}} \end{array}\right. } \end{aligned}$$

Given the above definition, we define the strategy $s_i$ of player *i* as a probability distribution over her set of actions *A*: $s_i \in S$, where *S* is the set of all possible strategies, and consists of the set of all possible probability distributions over the set of actions. A strategy profile $\varvec{s}$ is then a tuple containing the chosen strategies of all the agents $\varvec{s} = (s_1, \ldots , s_n) \in S^n.$ A strategy is called pure when the player has probability 1 of taking a specific action, and is called mixed otherwise. We refer to a profile $\varvec{s}$ where each agent selects *C* (respectively, *D*) with probability 1 as the *fully cooperative* (respectively, *fully competitive*) profile. Using the profile vector *s*, we can then define the expected utility as:

3 $$\begin{aligned} u_i(\varvec{s}, f, \varvec{c}) = \sum _{\varvec{a} \in A^n} u_i(\varvec{a}, f, \varvec{c}) \prod _{j=1}^n s_j(a_j) \end{aligned}$$

We furthermore use the notation $s_{-i} = (s_1, \dots , s_{i-1}, s_{i+1}, \dots , s_n)$ to denote the strategy profile obtained from $\varvec{s}$ by removing $s_i$. Analogously, we denote by $S_{-i}$ the set of such profiles.

For conciseness, hereafter we often assume the values of *f* and $\varvec{c}$ are fixed and write $u(\varvec{s})$ instead of $u(\varvec{s}, f, \varvec{c})$.

***Equilibria and optimal profiles***

Our starting point in this paper is the analysis of PGGs enabled by standard equilibrium theory [40]. We start by introducing the notion of dominant strategy and dominant strategy equilibrium.

#### Definition 2

(*Dominance*) Let a PGG be given. For any two strategies $s_{i}, {s_{i}}^{\prime } \in S$ of player *i*, we say that:

- $s_i$ dominates $s^{\prime }_{i}$ if $u_i(s_i, s_{-i}) \ge u_i(s^{\prime }_{i}, s_{-i})$ $\forall$ $s_{-i} \in S_{-i}$, and there exists $s^{* }_{-i} \in S_{-i}$ such that $u_i(s_i, s^{*}_{-i}) > u_i(s^{\prime }_{i}, s^{*}_{-i})$.
- $s_i$ weakly dominates $s^{\prime }_{i}$ if $u_i(s_i, s_{-i}) \ge u_i(s^{\prime }_{i}, s_{-i})$ $\forall$ $s_{-i} \in S_{-i}$.

A strategy is said to be (weakly) dominant for agent *i* if it (weakly) dominates all the other strategies of *i*.

#### Definition 3

(*Dominant strategy equilibria*) Given a PGG, a strategy profile $\varvec{s}$ is a (weakly) *dominant strategy equilibrium* if $\; \forall \; i \in N$, $s_i$ is a (weakly) dominant strategy for *i*.

Dominant strategy equilibria are refinements of Nash equilibria:

#### Definition 4

A strategy profile $s^{*} \in S^n$ is a *Nash equilibrium* (NE) if for every agent $i \in N$, the strategy $s^{*}_i$ is a best response to $s^{*}_{-i}$. That is, $u_i(s^{*}_i, s^{*}_{-i}) \geq u_i(s_i, s^{*}_{-i})$ for all strategies $s_i \in S_i$.

The mixed-motives characteristic of PGG we mentioned above, which is the focus of this paper, consists formally in the fact that PGG equilibria may be suboptimal in a very precise sense:

#### Definition 5

(*Pareto optimality*) Given a PGG, a strategy profile $\varvec{s}$ Pareto dominates strategy profile $\varvec{s}'$ if, $\forall \; i \in N$, $u_i(\varvec{s}) \ge u_i(\varvec{s}')$ and $\exists \; i \in N$ such that $u_i(\varvec{s}) > u_i(\varvec{s}')$. A strategy profile $\varvec{s}^{*}$ is Pareto optimal if there is no strategy profile $\varvec{s}$ that Pareto dominates it.

***Analysis in pure strategies***

To set the stage for introducing our novel environment in the next section, it is useful to provide a simple fact about rational choices in the Public Goods Game as we defined it above. In particular, we can observe that the value of the multiplication factor *f* fully determines which one between *C* or *D* is a dominant strategy.

#### Proposition 1

For any PGG $\langle N, \varvec{c}, A, f, \varvec{u} \rangle$, let $\varvec{s}_C$ and $\varvec{s}_D$ denote the fully cooperative and fully competitive profiles, respectively. Then:

(i) If $0 \le f \le 1$, $\varvec{s}_D$ is Pareto optimal and the only dominant strategy equilibrium.
(ii) If $1<f\le n$, then $\varvec{s}_C$ is Pareto optimal, and $\varvec{s}_D$ is a weakly dominant strategy equilibrium.
(iii) If $f>n$, $\varvec{s}_C$ is Pareto optimal and the only dominant strategy equilibrium.

#### Proof

See Appendix D. $$\square $$

**Fig. 2:**
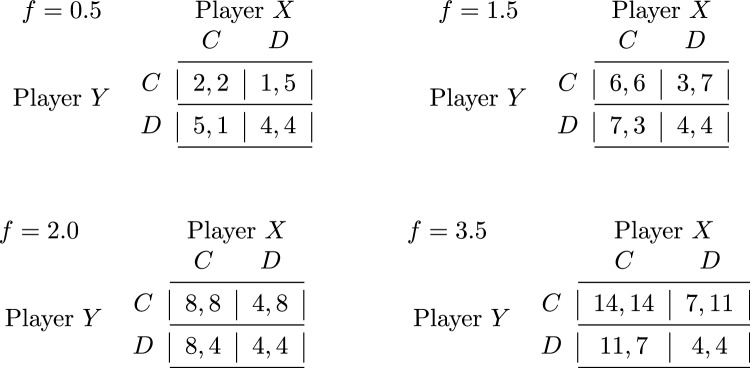
Normal form games instantiating the PGG for two players (X and Y) with 4 coins each, for four possible values of the multiplication factor: $f=0.5$, that is, a competitive game where *DD* is a dominant strategy equilibrium that is also Pareto dominant; $f=1.5$, that is, a mixed-motive game where *DD* is a dominant strategy equilibrium but it is Pareto dominated by *CC*; $f=2.0$, that is, a boundary mixed-motive game where *DD* is now a weakly dominant strategy equilibrium, again Pareto dominated by *CC*; $f=3.5$, that is, a cooperative game where *CC* is a dominant strategy equilibrium that is also Pareto dominant

Intuitively, the proposition follows from simple observations. The best-case scenario for agent *i* is the profile where all other players invest, generating a public good value of $f \cdot \sum _{j\ne i} c_j \cdot (n-1)$. In such case, if $f > n$, then the dominant strategy for *i* is to contribute to the production of the good, by Equation (1). If $f \le n$, then it is (weakly) better for *i* to defect, and this remains the case through to the worst-case scenario in which every other player defects. However, public goods whose value exceeds their costs can be produced whenever $f > 1$. In such cases, full cooperation is Pareto optimal. An example of Proposition 1 is given in Fig. 2, which illustrates the normal form representation of the game for two players using the multiplication factors $f=\{0.5,1.5,2.0,3.5\}$.

### The extended public goods game

The classic Public Goods Game studied in game theory and behavioural economics focuses on the interval in which the multiplication factor is bigger than 1 and smaller than the number of players $1<f<N$. However, in this work we want our agents to be able to cope with a spectrum of environments spanning from cooperative, to competitive, to mixed-motive. Therefore, we extended the interval of allowed multiplication factors to $0<f<R_{+}$, where $R_{+}>N$ is an arbitrary value, that enables the agents to play in cooperative settings too. The range $f<1$ allows us to face competitive scenarios as well. We call this setting the Extended Public Goods Game (EPGG). Varying the multiplication factor *f* can define a set of environments on which we train our RL agents. At every interaction among the agents, a different environment is sampled from the set of available ones. In these environments, the agents have access to information regarding the amount of coins they are endowed with and the value of the multiplication factor. With these observations, we expect agents to learn to cooperate when the multiplication factor is bigger than the number of players and to learn to defect with a certain probability when the value is smaller.

The main objective of our work is to study the effect of emergent communication on uncertain observations when agents are trained on a set of cooperative, competitive and mixed environments. In particular, we want to observe the effects of these ingredients when the uncertainty is imposed on the degree of alignment of incentives. For this purpose, we introduce the possibility of observing the multiplication factor with uncertainty.

## Methods

In this section, we describe the framework we adopt to tackle the learning process in the EPGG setting, i.e. multi-agent reinforcement learning (MARL), and the architectures we use, delving into the implementation of how agents deal with uncertainty and the communication protocol.

### Multi-agent reinforcement learning

When mapping the EPGG to the MARL framework, in this case a partially observable stochastic game, we structure the interactions in the following manner. At the beginning of each episode *t* (i.e. interaction), a multiplication factor $f_t$ is sampled from a given set $F = \{f_0, \dots , f_K\}$ of factors, where *K* is the total number of available values (for the specific values chosen for this set see Sect. 5). The observation $o_{i,t}$ received by agent *i* (with $i=0, \dots , n$) in episode *t* is specified by the sampled multiplication factor and the endowment $c_{i,t}$, so $o_{i,t} = (f_t, c_{i,t})$. In our experiments, we fix the agents’ endowments to the same constant value $c_{i,t} = c$, thereby assuming all agents have the same endowment to invest in public goods provision.

After $o_{i,t}$ has been observed, agents choose an action (*C* or *D*) and receive a reward $r_{i,t}$ as determined by the definition of the EPGG (Eq. 1). The episodes have length 1. Under no uncertainty, the agents receive the precise value of the multiplication factor $f_t$; otherwise, they observe it with added noise, $\hat{f}_{t}$ (Sect. 4.2). Note that this feature implies a continuous state space. To this end, we opt for RL methods incorporating neural function approximation.

We study distinctly two scenarios: the one without communication among agents (detailed below) and the one with communication (Sect. 4.3).

In the setting without communication, after receiving the observation, all the agents act simultaneously. The input of the action policy coincides with the received observation of agent *i*: $\pi _{A_i} :O_i \times A \rightarrow [0,1]$. After acting, every agent receives a reward $r_{i,t}$ from the environment, which is equal to the utility function of the EPGG presented in Eq. 1, and therefore depends on the current value of the multiplication factor $f_t$, the endowment $\varvec{c}_t$ and the current joint action of the agents $\varvec{a}_t :r_{i,t} = u_{i,t}(\varvec{a}_t, f_t, \varvec{c}_t)$. Since different scenarios bring different rewards, we normalized the rewards between 0 and 1. The normalization of the reward for the agents playing in a specific scenario is computed by dividing the current reward received by the maximum possible reward the agent could have received in that scenario.

In this work, agents are independent learners and are trained using two distinct algorithms: a policy-based algorithm and a value based one, to validate the consistency of the results among different reinforcement learning approaches. Those are, respectively, the REINFORCE algorithm with baseline [60] and the deep Q-network (DQN) algorithm [45]. All the networks are implemented as multi-layered perceptrons with one or two hidden layers and *tanh* nonlinearities. In the following, we give a brief overview of the two algorithms.

***REINFORCE***

Given any differentiable policy $\pi (a|s,\theta )$, where $\theta$ denotes the parameters of the policy (i.e. the weights of the neural network), the objective function of the REINFORCE algorithm with baseline is defined as follows:

4 $$\begin{aligned} J(\theta ) = \int p_{\theta }(\tau ) ( G(\tau ) - b ) \textrm{d}\tau , \end{aligned}$$

where $\tau$ is a variable denoting the trajectory which, in an episodic environment such as ours, is a finite length sequence $s_1, a_1, r_1,..., s_T, a_T, r_T$ of state–action–reward triplets; $p_{\theta }(\tau )$ is the probability of encountering trajectory $\tau$ and depends on the parameter vector of the policy $\theta$; $G(\tau )$ is the sum of the rewards obtained by the agent in the trajectory $\tau$, $G(\tau ) = \sum _{i=1}^T r_i$; and finally, *b* is called baseline and is a quantity that allows us to reduce the variance of the update. The weights of the neural network are updated by stochastic gradient ascent as:

5 $$\begin{aligned} \theta _{t+1} \leftarrow \theta _{t} + \alpha \nabla _{\theta } J(\theta _t), \end{aligned}$$

where $\alpha$ is the learning rate and $\nabla _{\theta } J(\theta _t)$ is the gradient of the objective function. Using the policy gradient theorem [56], the above can be written as:

6 $$\begin{aligned} \nabla _{\theta } J(\theta ) = \mathop {\mathbb {E}}_{\tau \sim p_{\theta }(\tau )} \Big [ \sum _{t=1}^T \nabla _{\theta } \log \pi _{\theta }(a_t|s_{t}) (G(\tau ) - b)\Big ] \end{aligned}$$

An estimator of the gradient of the objective function that is often used is:

7 $$\begin{aligned} \widehat{\nabla }_{\theta } J(\theta ) = \mathop {\mathbb {E}_t} \Big [ \nabla _{\theta } \log \pi _{\theta _t}(a_t|s_{t}) (G_t(\tau ) - b_t)\Big ] \end{aligned}$$

where the expectation is computed over the sample of trajectories produced at the learning step *t* by policy $\pi _{\theta _t}(a_t|s_{t})$. Given a sample of size *n*, the baseline $b_t$ is computed as the average of the rewards in the sample:

8 $$\begin{aligned} b_t = \frac{1}{n} \sum _{i=0}^n r_i \end{aligned}$$

***Deep Q-Network (DQN)*** The DQN algorithm is the natural function approximation extension of the tabular Q-learning [59] approach. In DQN, the Q-function is represented using a neural network parameterized by $\theta$: $Q(s,a;\theta ) \sim Q^{*}(s,a)$, where $Q^{*}(s,a)$ is the optimal value function. This approximation is achieved by minimizing the mean squared error between the temporal difference target and the current Q-function estimate:

9 $$\begin{aligned} L({\theta }) = \mathbb {E}_{s_t, a_t, r_t, s_{t+1} \sim U(D)}[(r_t + \gamma \text {max}_{s \in A} Q(s_{t+1},a; \theta ^{-}) - Q(s_{t+1},a; \theta ))^2] \end{aligned}$$

The expectation is computed over a batch of trajectories sampled uniformly from the experience memory buffer *D*. The vector of parameters $\theta ^{-}$ contains the value of the network weights some iterations *k* before the current update. This network is called the target network and allows to stabilize the learning. The agent’s policy during training is $\epsilon -$greedy:

10 $$\begin{aligned} \pi (a|s) = {\left\{ \begin{array}{ll} (1-\epsilon ) + \epsilon /|A| &  \quad \text { if } a = \text {argmax}_{a \in A} Q(s,a,\theta )\\ \epsilon /|A| &  \quad \text { otherwise} \end{array}\right. } \end{aligned}$$

The final policy is defined as the one that selects actions greedily based on the estimated Q-function values.

In our work, we evaluated both the REINFORCE and DQN algorithms in two-agent experiments, but in the main text of the paper, we will focus on the results obtained by employing the REINFORCE algorithm. We perform experiments with the DQN algorithms as well to ensure consistency of the results among different RL approaches. Where relevant, we will report and comment also on the results obtained using the DQN algorithm. All results concerning the DQN algorithm can, however, be found in Appendix B. Our choice is mainly motivated by the fact that the REINFORCE algorithm aligns with the previous literature on emergent communication, but also due to the faster convergence of the REINFORCE algorithm, particularly in complex scenarios that involve training communication and action policies simultaneously.

### Uncertainty

In the EPGG, uncertainty is introduced as Gaussian noise over the observation of the multiplication factor: the observed $\hat{f}_{i,t}$ is randomly sampled from the distribution $\hat{f}_{i,t} \sim N(f_t, \sigma _i)$, where $f_t$ is the multiplication factor of the environment at training step *t* and $\sigma _i$ is the uncertainty of agent *i*.

When uncertainty is present, we allow agents to handle it with or without using a model. Without a model, the noisy observation is directly provided as part of the agents’ input, and we let the neural networks to inherently model the uncertainty. Otherwise, agents keep a probabilistic model of the observed factors via a Gaussian mixture model (GMM).^2^ This model allows agents to maintain a belief over the observed multiplication factor, under uncertainty. Agents keep a history of all the multiplicative factors observed during the training to fit a GMM. Afterwards, the vector of predicted probabilities for each component of the mixture is provided as part of the input to the agent’s network.

### Communication

In the communication scenario, before acting, a subset *Z* of agents (hence, $0 < |Z| \le n$) sends a message sampled from the output layer of their communication network. The observations received from the environment define the input of the communication policy $\pi _{C_i}:O_i \times M \rightarrow [0,1]$ where *M* is the set of possible messages, whose size is a training hyperparameter. Basing ourselves on previous work in emergent communication, we choose to work with a discrete message set. This choice is based on the idea that a discrete policy could be more naturally interfaced with natural language [27, 35]. After receiving the observation from the environment, the communicating agent *i* sends a message $m_{i,t}$. The messages of all the communicating agents are concatenated, and represent, together with the observations, the input to the action policy $\pi _{A_i}:O_i \times M^{|Z|} \times A \rightarrow [0,1]$. From this point onwards, the episode proceeds as in the non-communicative scenario. Algorithm 1 presents the full learning algorithm employed in the case of communication.

To facilitate the agents to use the communication channel, we modify the loss functions of the communication and action policies as suggested by Eccles et al. in [20]. The goal is to bias the agents towards positive signalling, encouraging them to send distinct messages in response to different observations, and positive listening, encouraging agents to respond differently when different messages are received. Considering a communication policy $\pi _{\theta _C}$, where $\theta _C$ is the parameter vector of the communication policy, and observation $o_t$, we define a loss term which is minimized when the average entropy of the message policy is high $H(\overline{{\pi }_{\theta _C})}$, and the entropy of the message policy conditioned on the input $H(\pi _{\theta _C}|o_t)$ reaches a target value $H_{target}$ (a hyperparameter whose value has been set to $\log |A|/2$):

11 $$\begin{aligned} L_{ps}(o_t,\theta _C) = - \mathop {\mathbb {E}_t}[\lambda H(\overline{{\pi }_{\theta _C})} - (H(\pi _{\theta _C}|o_t)-H_{target})^2], \end{aligned}$$

where the expectation is computed over the sample of trajectories produced at the learning step *t* and $H(\overline{{\pi }_{\theta _C})}$ is estimated over the messages in the batch. The complete loss used for the communication policy is a weighted combination of the REINFORCE loss and the loss for positive signalling:

12 $$\begin{aligned} L_{C}(o_t, \theta _C) = \mathop {\mathbb {E}_t} \Big [ \log \pi _{\theta _C}(a_t|o_{t}) (G_t(\tau ) - b_t)\Big ] + \lambda _C L_{ps}(o_t,\theta _C) \end{aligned}$$

To foster positive listening, we add a term to the loss of the action policy, which is maximized when the actions of an agent are highly influenced by the messages they receive. Considering an action policy $\pi _{\theta _A}$, where $\theta _A$ is the parameter vector of the action policy, and observation $o_t$, we compute the divergence between the agent’s policy conditioned on the received message $m_t$, and the unconditioned one, where the message is replaced by the empty vector $\varvec{0}$:

13 $$\begin{aligned} L_{pl}(o_t,m_t,\theta _A) = - \sum _{a \in A} |\pi _{\theta _A}(a|o_t,m_t) - \pi _{\theta _A}(a|o_t,\varvec{0})| \end{aligned}$$

where $m_t$ is the concatenated vector of the messages received from all the agents at time step *t*, $\varvec{0}$ is the empty vector with the same size of $m_t$ and *A* is the set of possible actions.

The complete loss used for the action policy is a weighted combination of the REINFORCE loss and the loss for positive listening:

14 $$\begin{aligned} L_{A}(o_t, m_t,\theta _A) = \mathop {\mathbb {E}_t} \Big [ \log \pi _{\theta _A}(a_t|o_{t},m_t) (G_t(\tau ) - b_t)\Big ] + \lambda _A L_{pl}(o_t, m_t,\theta _A). \end{aligned}$$

The losses of the DQN algorithm have been modified in the same way.

In order to quantify the information exchanged by the agents, we implement measures to detect signalling and listening behaviours, following the methods proposed by [43]. In particular, we measure the mutual information, which quantifies the correlation between messages and actions. Given a message policy and an action policy, the mutual information between messages and actions is:

15 $$\begin{aligned} MI = \sum _{a \in A} \sum _{m \in M} p(a,m) \log \frac{p(a,m)}{p(a) p(m)}, \end{aligned}$$

where, we recall, *A* is the set of actions available to the agents and *M* is the set of messages. Probabilities are computed empirically during training, by averaging over the messages and actions of the epoch. If messages and actions come from the same agent, this measure takes the name of *speaker consistency*, and allows us to determine how much the actions and the messages of the agent are aligned. If instead, they come from different agents, we are observing how much the messages sent by one agent influence the actions taken by another. In this case, the metric takes the name of *instantaneous coordination*, and it measures positive listening for the agent that takes the actions [43].

Algorithm 1
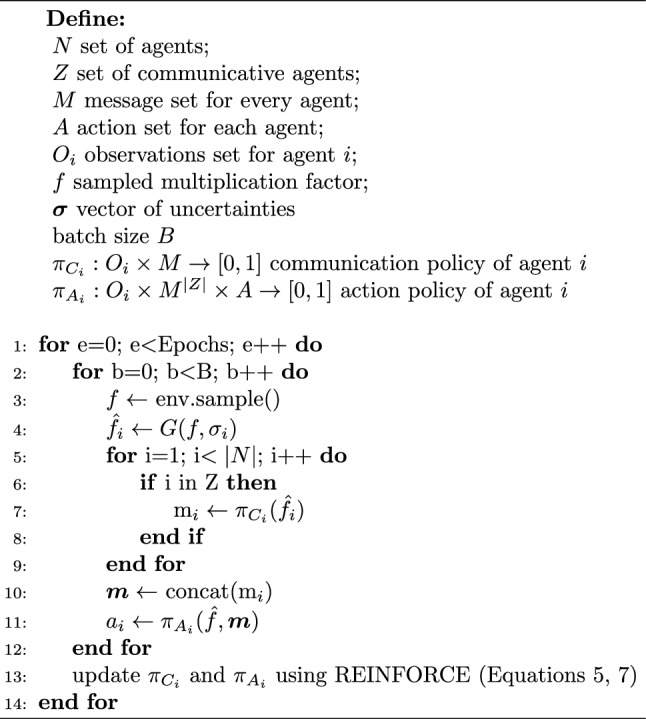


## Results

Our objective is to analyse the behaviour of reinforcement learning agents trained on a pre-specified set of multiplication factors that define cooperative, competitive and mixed scenarios, and in the presence of uncertainty about this factor and, therefore, the level of (mis-)alignment of their incentives. We implemented the EPGG environment using the PettingZoo library [57]. Our environment is available on GitHub, together with the experiments.^3^ In the experiments, the values of the multiplication factor have been chosen so that in the scenarios without incentive uncertainty the agents converge to the dominant strategies. This approach ensures that simultaneous training across a spectrum of environments does not bias the learning in any specific environment.

In all the experiments, the value of the endowment is fixed to 4 coins for all the agents. In every scenario involving incentive uncertainty, experiments are run both with and without the GMM module, which is applied to the uncertain agents only. The presented outcomes result from averages of 80 experiments on each considered scenario. The main results of the experiments, displayed in terms of average cooperation of the agents at the end of the training, are reported in Table 1a and 1b for the REINFORCE algorithm and in Table 2 for the DQN algorithm. Statistical *t*-testing has been performed to assess the significance of the difference between the experiments with and without communication, and the outcomes of the tests are reported in Appendix A. The hyperparameters employed in the experiments are described in Appendix C.

### Two-player games

In the scenario consisting of two players, we define the following set of multiplication factors: $F = \{ 0.5, 1.5, 2.5, 3.5 \}$. In this game, for rational agents, it is dominant to defect when $f \in \{0.5, 1.5\}$ and to cooperate when $f \in \{2.5, 3.5\}$. Hereafter we refer to $f \in \{2.5, 3.5\}$ as cooperative games, $f=0.5$ as the competitive game and $f=1.5$ as the mixed-motive one. We perform experiments for three different training scenarios: (1) both agents have full observability of the environment; (2) one agent receives uncertain observations of the multiplication factor; and (3) both agents receive uncertain observations of the multiplication factor.

Below we discuss the main findings. Full details on the data generated and the analysis can be found in Appendix A. We emphasize that the results concern REINFORCE, as it aligns with the previous literature on emergent communication. We present plots illustrating the returns for the agents in the different scenarios. In the plots, we also add three baselines, displayed as horizontal lines: the returns the agents would obtain if they always cooperate (dashed red line), always defect (dashed blue line), cooperate or defect with probability 0.5 (dashed green line).

**Fig. 3:**
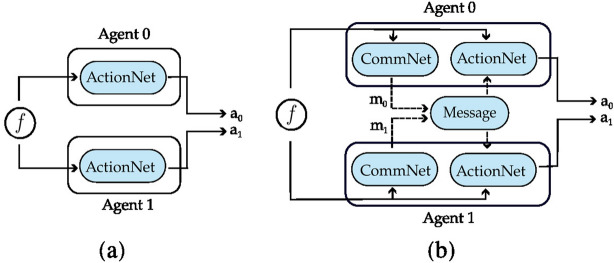
Setting deployed for the two-agent experiments with one uncertain agent (agent 1). **a** Settings with the action policy only, **b** setting with both the communication and action policies

**Fig. 4:**
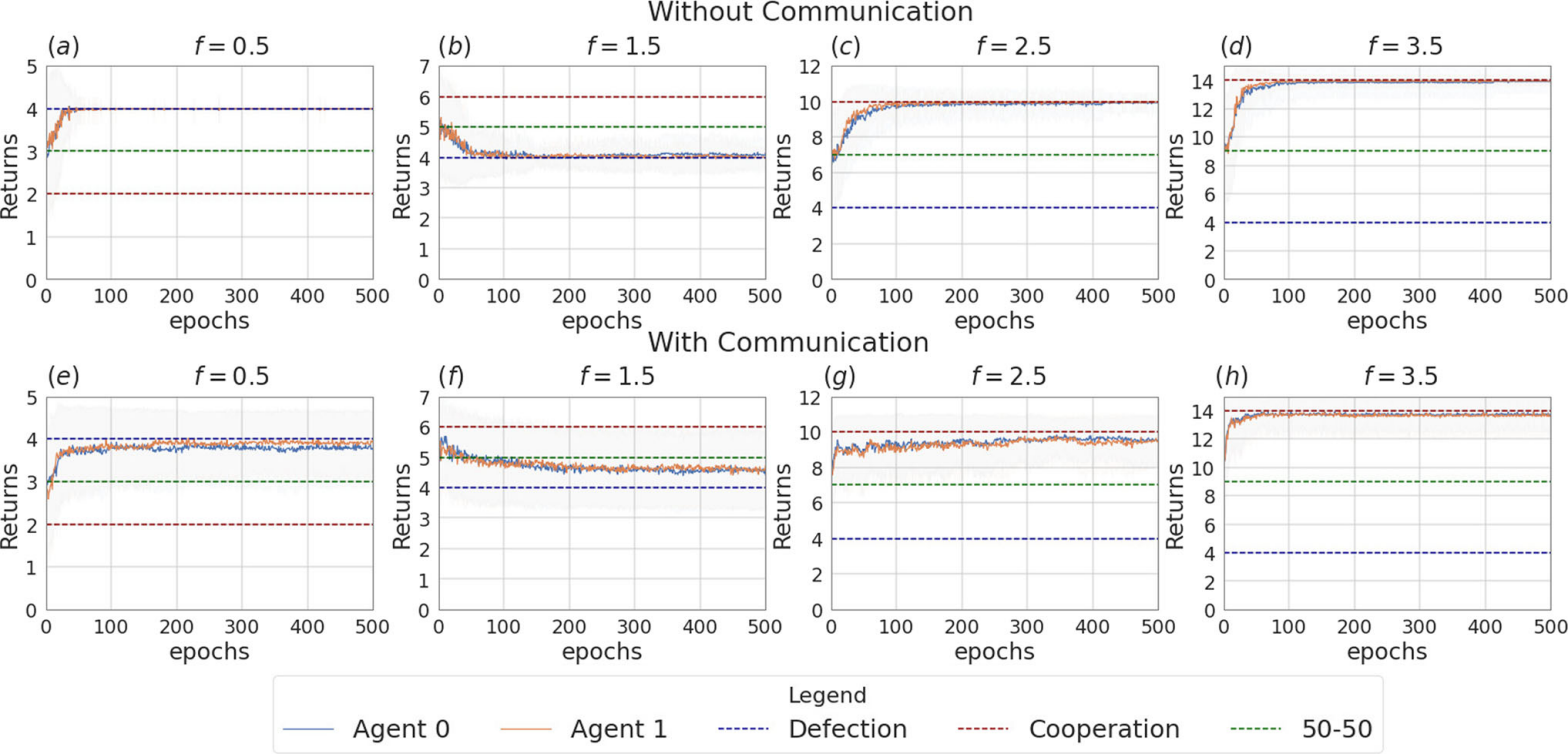
Returns during training for the setting with two agents and without incentive uncertainty, in the scenarios without communication (top row) and with communication (bottom row). The curves are averages over 80 runs. The horizontal dashed lines represent the returns the agents would obtain if they always cooperate (dashed red line), always defect (dashed blue line), cooperate or defect with probability 0.5 (dashed green line). Agents are trained using REINFORCE

**Fig. 5:**
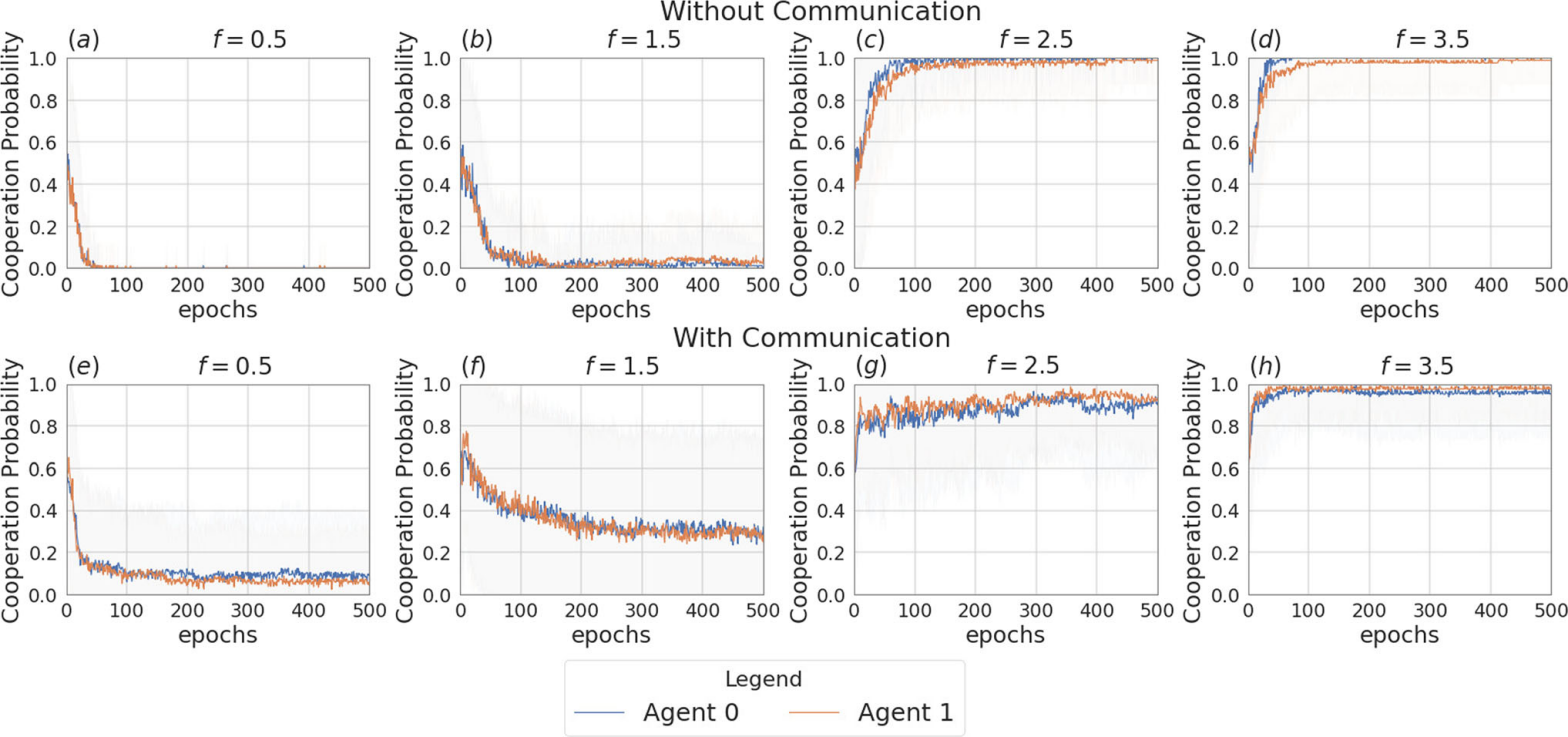
Probability of cooperating during training for the setting with two agents and no incentive uncertainty, in the non-communication case (upper row) versus communication case (lower row). Agents are trained using REINFORCE

Figure 3 presents the two scenarios employed for training agents when no incentive uncertainty is introduced, both without (image (a)) and with communication (image (b)) among the learning agents. Fig. 4 shows the returns during training, per each multiplication factor. In the communication scenario, both agents send and receive messages. The results for the setting with and without communication are consistent for the cooperative and competitive games: in the cooperative games, the agents converge to the cooperative action, which is the strictly dominant strategy equilibrium and Pareto optimal profile, and allows to maximize the benefit of the whole group. The same happens when $f = 0.5$, where as expected the agents converge to defection. When faced with the mixed-motive game, the non-communicating agents converge to the defection as well, while in the communication scenario, the probability to cooperate reaches an average of 0.32: here the social welfare of the group (i.e. the sum of returns over all the agents) increases over the dominant strategy profile. The agents therefore avoid full defection but still fail to reach full cooperation. This result can be observed in Fig. 5, which shows the evolution of the average cooperation probability of the agents during training. The Welch’s *t*-test has been performed to confirm the significativity of these observations. When the REINFORCE algorithm is employed, we observed no significant difference between the scenarios with and without communication when $f \in \{0.5, 2.5, 3.5\}$ when performing the *t*-test with a *p* value threshold of 0.001. The difference between the averages is significant only when $f=1.5$ (see Table 3). When using the DQN algorithm, no significant difference has been detected (see Table 4).

**Fig. 6:**
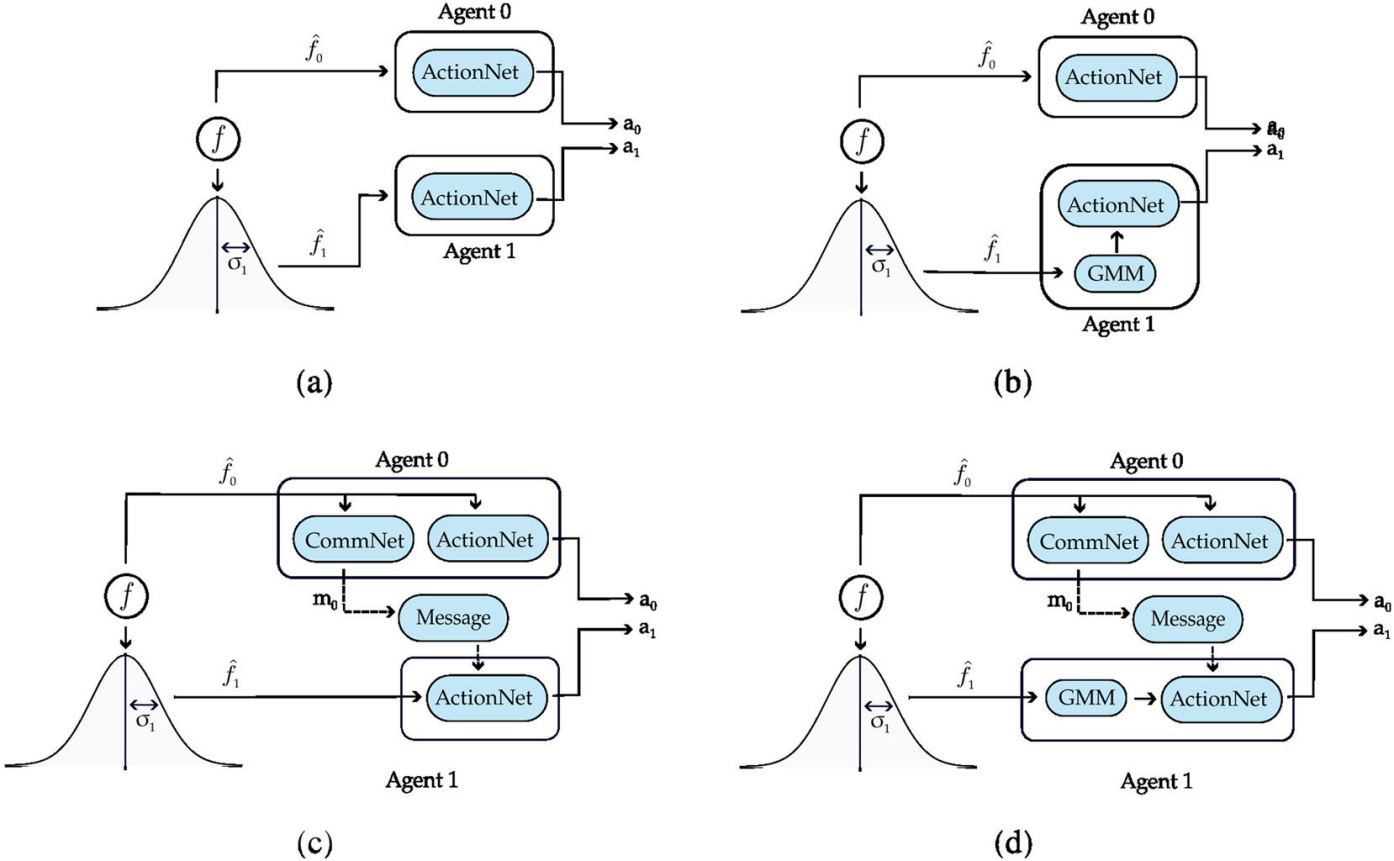
Setting deployed for the two-agent experiments with one uncertain agent (agent 1). **a** Scenario with the action policy only and no uncertainty modelling, **b** scenario with the action policy and uncertainty modelling over agent 1 via GMM, **c** scenario with the action and communication policies and no uncertainty modelling and **d** scenario with the action and communication policies and no uncertainty modelling and uncertainty modelling over agent 1 via GMM

**Fig. 7:**
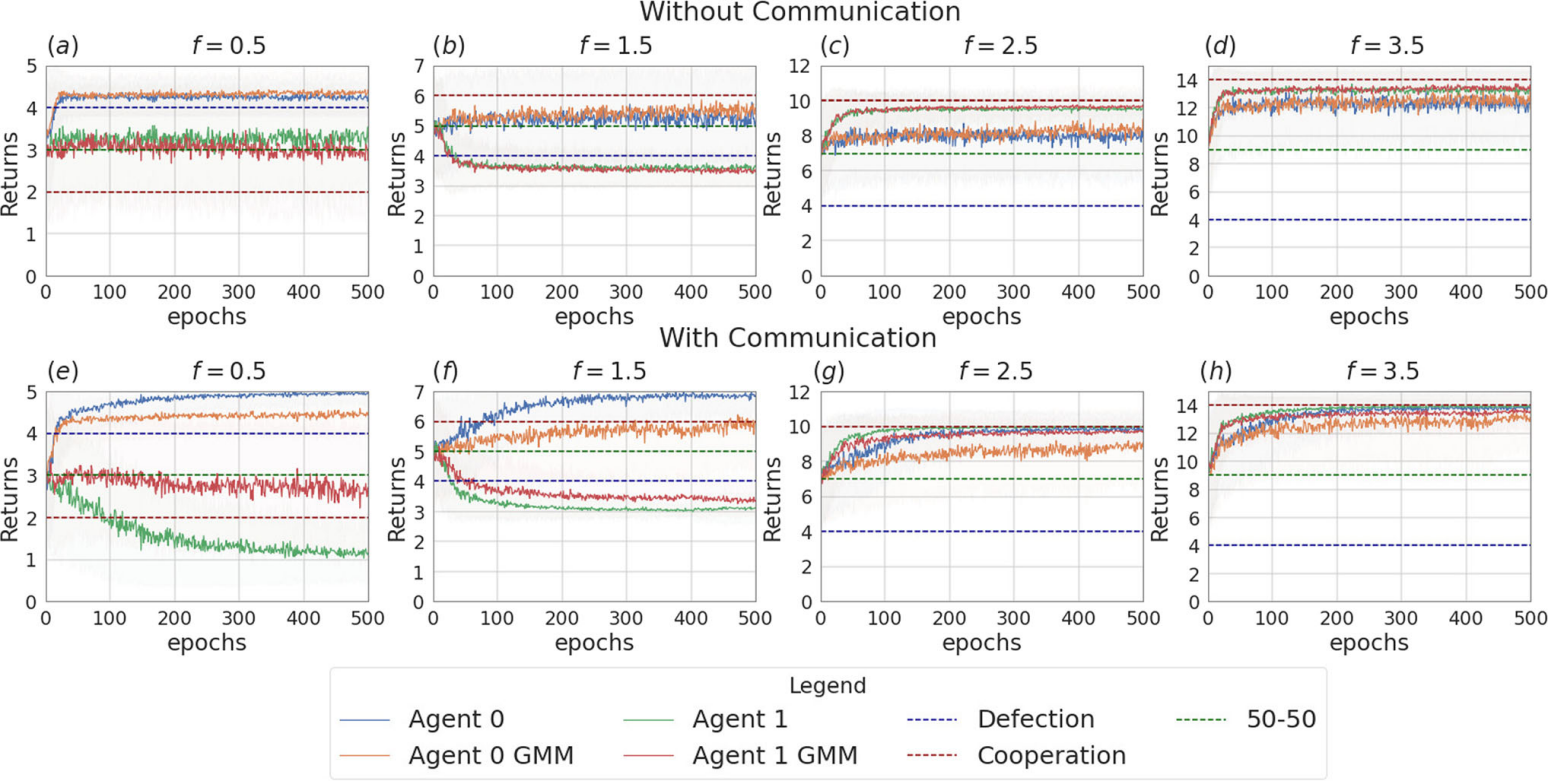
Returns during training, in the scenario with one uncertain agent (agent 1, with $\sigma =2$), in the non-communication case (upper row) versus communication case (lower row). The curves are averaged over 80 runs. The horizontal dashed lines represent the returns the agents would obtain if they always cooperate (dashed red line), always defect (dashed blue line), cooperate or defect with probability 0.5 (dashed green line). Agents are trained using REINFORCE (Color figure online)

**Fig. 8:**
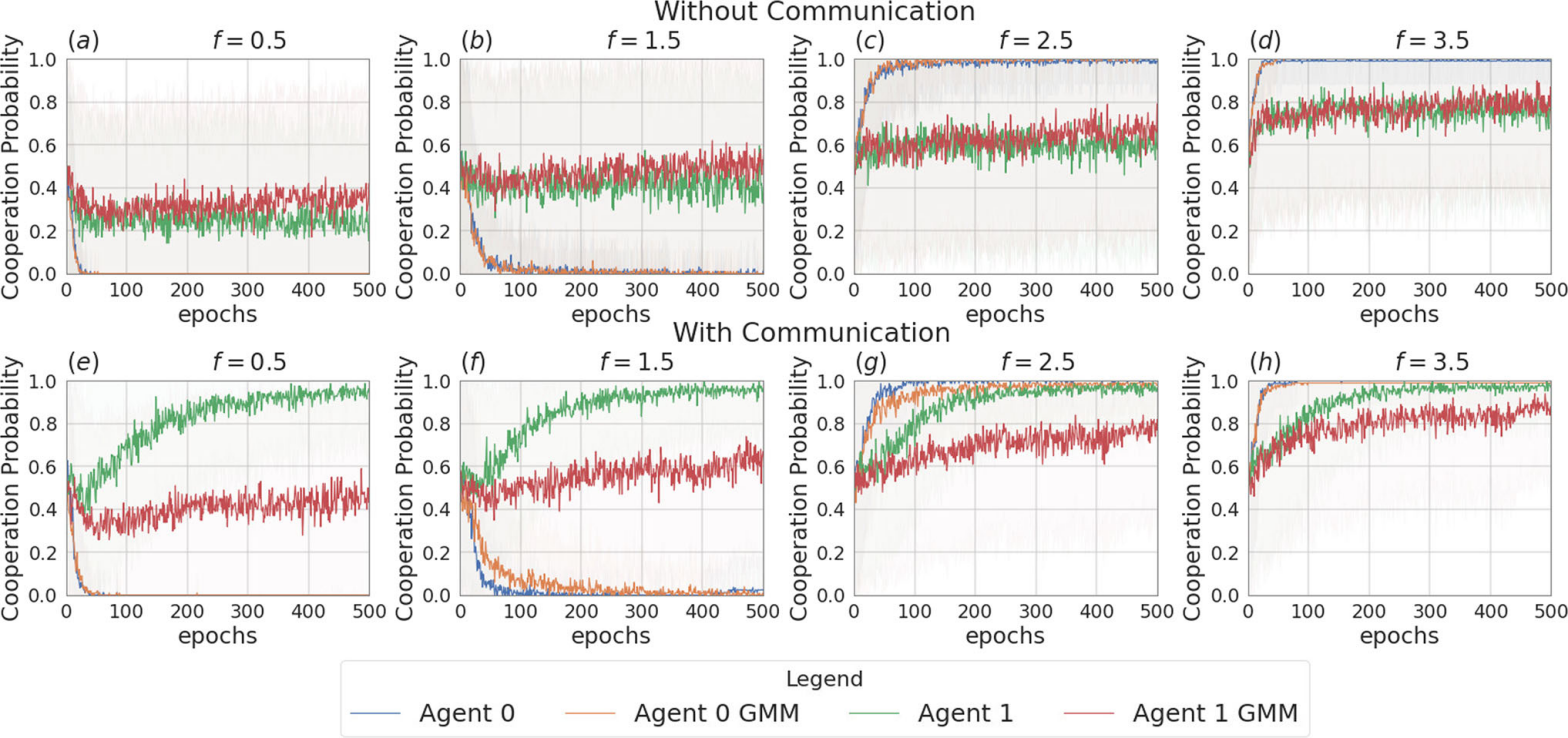
Probability of cooperating during training, in the scenario with one uncertain agent (agent 1, with $\sigma =2$), in the non-communication case (upper row) versus communication case (lower row). Agents are trained using REINFORCE

Figure 6 presents the four scenarios employed when one agent is uncertain (agent 1), both without (images (a) and (b)) and with communication (images (b) and (c)) among the learning agents, and both without (images (a) and (c)) and with (images (b) and (d)) GMM model for uncertainty. Figure 7 depicts the returns during training when agent 1 is uncertain, and no communication channel is introduced. The incentive uncertainty is fixed to $\sigma =2$. As expected, adding uncertainty over the observations of one agent worsens its performance: the agent cannot disentangle the situations in which it is preferred to cooperate from the ones in which it is preferred to defect. Modelling uncertainty in this case does not change the outcome. This can be observed in Fig. 8, which reports the evolution of the cooperation probability of the agents during training, when the REINFORCE algorithm has been employed: for the uncertain agent, the probability of cooperating does not converge to zero when $f \in \{0.5, 1.5\}$ and does not converge to one when $f \in \{2.5, 3.5\}$. In this scenario, the certain agent (agent 0), as expected, keeps converging to the rational behaviours. The same results have been found by employing the DQN algorithm (Fig. 17). We introduce a unidirectional communication step in this scenario, where the certain agent is allowed to send messages to the uncertain one. Employing the REINFORCE algorithm, we notice the following effects:

- in the cooperative games ($f \in \{2.5, 3.5\}$), communication helps the uncertain agent to recover the optimal strategy, raising the average cooperation probability from 0.61 to 0.97 in the first case and from 0.80 to 0.97 in the second one; in this scenario, modelling uncertainty does not provide any additional benefit to the cooperation probability values;
- in the competitive and mixed games ($f \in \{0.5, 1.5\}$), communication allows the certain agent to exploit the uncertain agent, considering the case in which no uncertainty modelling module is used: in these scenarios, the average cooperation probability of the uncertain agent moves from 0.17 to 0.95 in the first case and from 0.36 to 0.96 in the second one. The cooperation values rise also in the case in which the uncertainty modelling is used, but not in a statistically significant way (see *p* values in Table 7b). We argue that explicitly modelling the uncertainty of the environment can provide the agent with additional information, making her less susceptible to deception.

This second claim is supported as well by Fig. 12, which shows the trend of the instantaneous coordination and speaker consistency (Eq. 15) during training (in the three scenarios), for the agents trained using the REINFORCE algorithm. (The results concerning DQN are presented in Fig. 19.) We observe that the instantaneous coordination is higher when one agent is uncertain and no modelling over uncertainty is allowed (Fig. 12b): here, the only way to recover information is to listen to the received messages and act accordingly. However, the uncertain agent gets easily deceived in this way, as shown by the speaker consistency metric (i.e. agent 0 is not acting in line with its own messages). In the experiments conducted using DQN, the introduction of the communication channel does not bring any difference in the outcome, and the deception effect is not observed (Fig. 17).

**Fig. 9:**
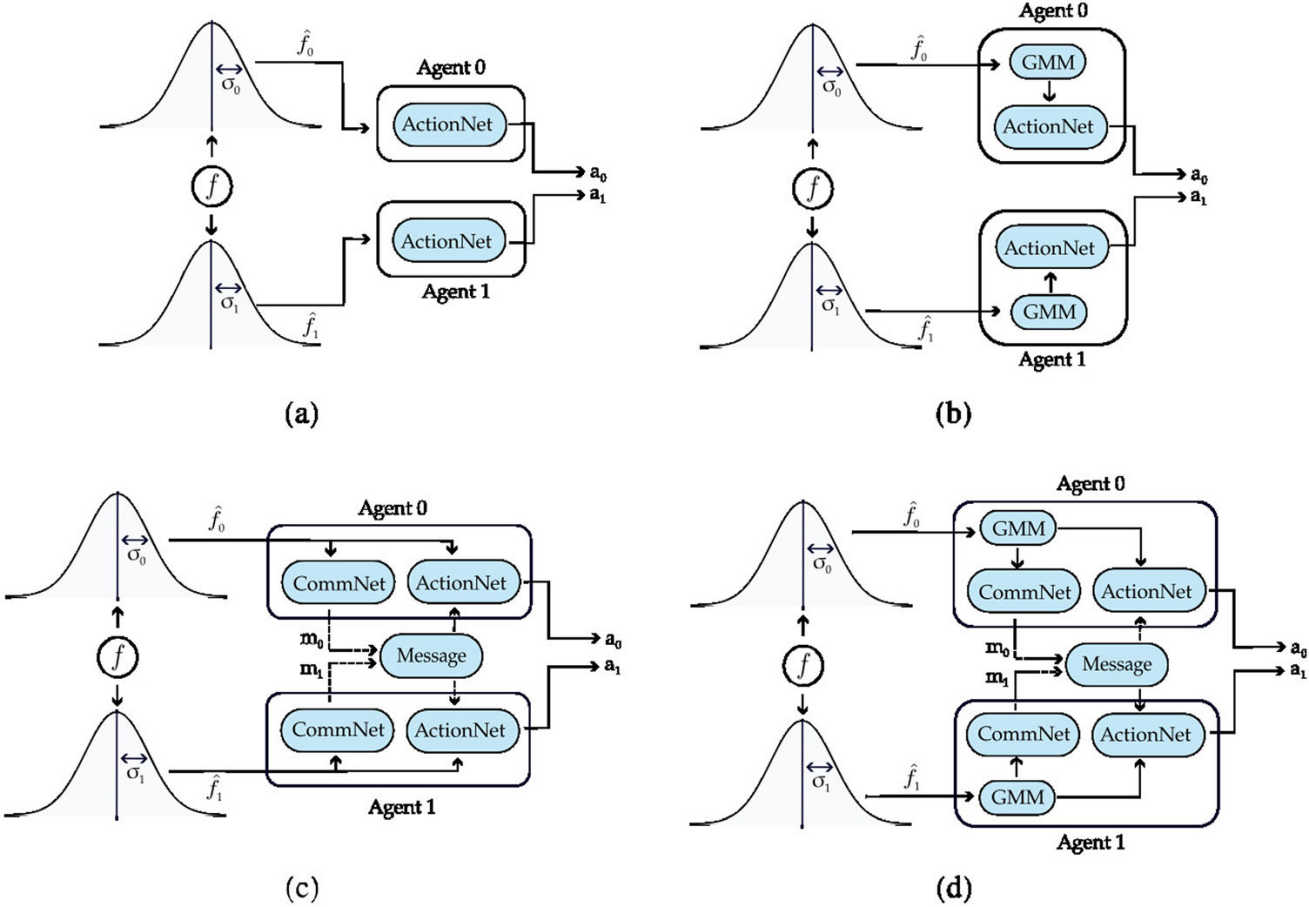
Setting deployed for the two-agent experiments with one uncertain agent (agent 1). **a** Scenario with the action policy only and no uncertainty modelling, **b** scenario with the action policy and uncertainty modelling via GMM, **c** scenario with the action and communication policies and no uncertainty modelling and **d** scenario with the action and communication policies and no uncertainty modelling and uncertainty modelling via GMM

**Fig. 10:**
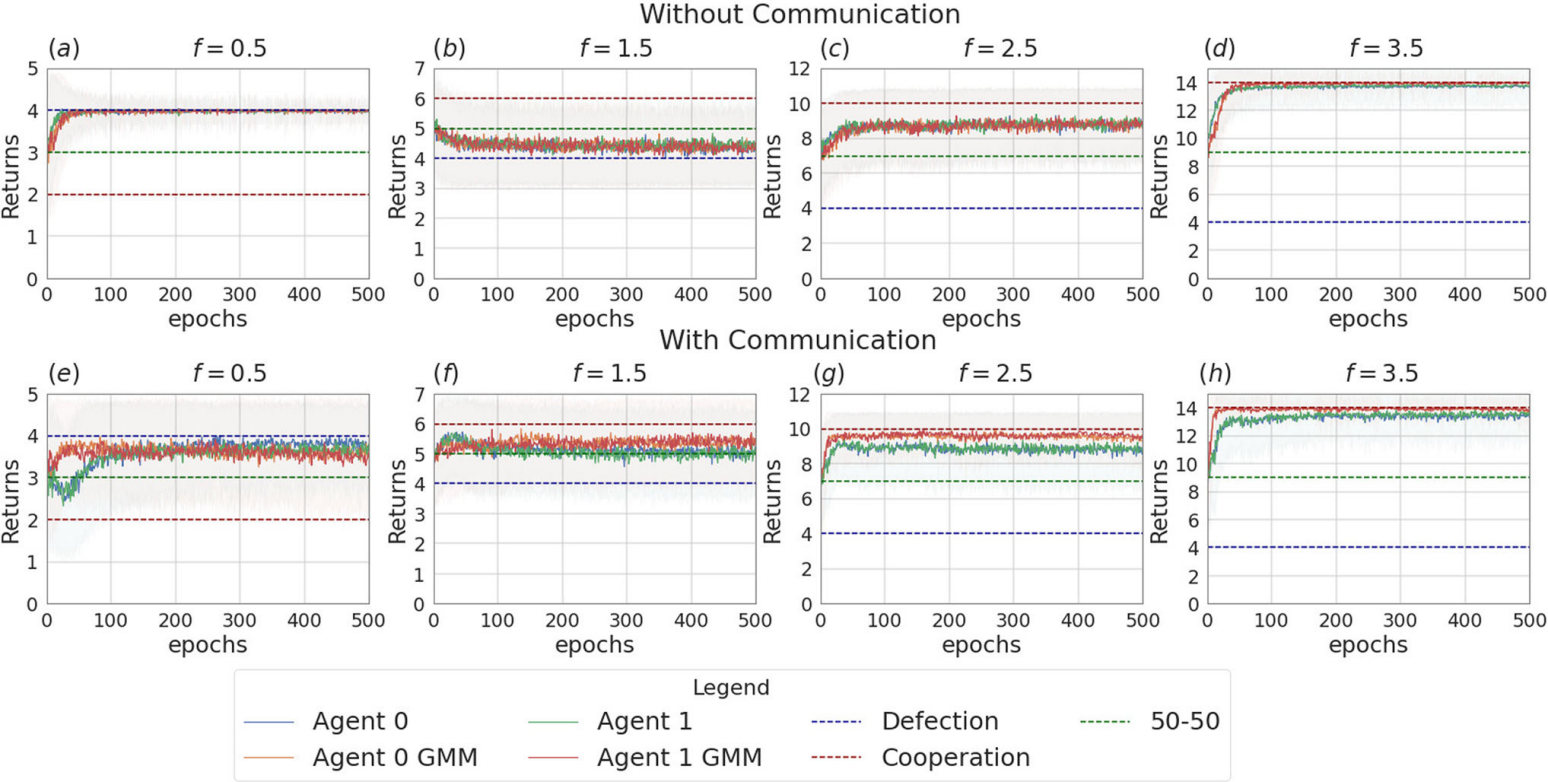
Returns during training, in the scenario with two uncertain agents ($\sigma =0.5$), in the non-communication case (upper row) versus communication case (lower row). The horizontal dashed lines represent the returns the agents would obtain if they always cooperate (dashed red line), always defect (dashed blue line), cooperate or defect with probability 0.5 (dashed green line). Agents are trained using REINFORCE

**Fig. 11:**
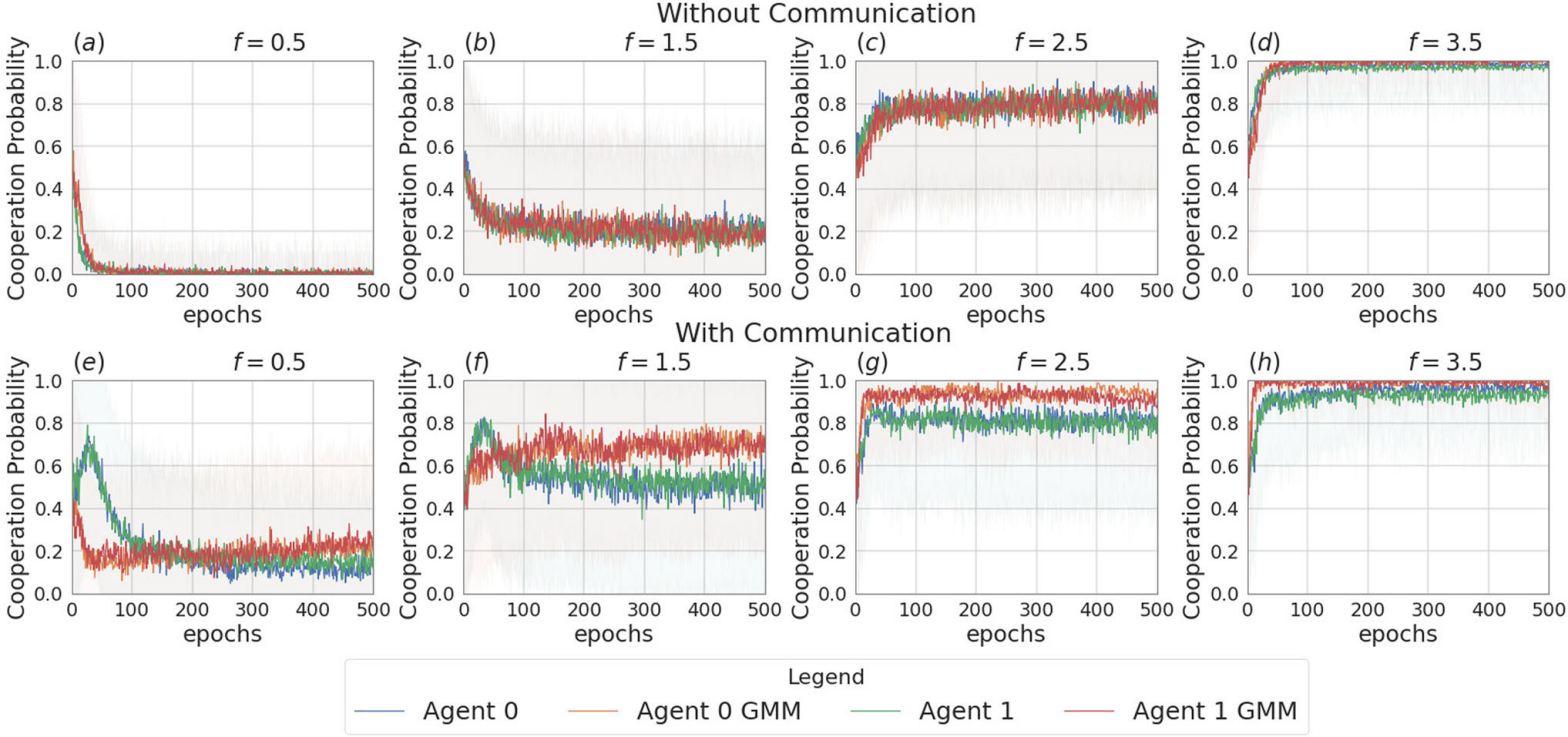
Probability of cooperating during training, in the scenario with two uncertain agents ($\sigma =0.5$), in the non-communication case (upper row) versus communication case (lower row). Agents are trained using REINFORCE

**Fig. 12:**
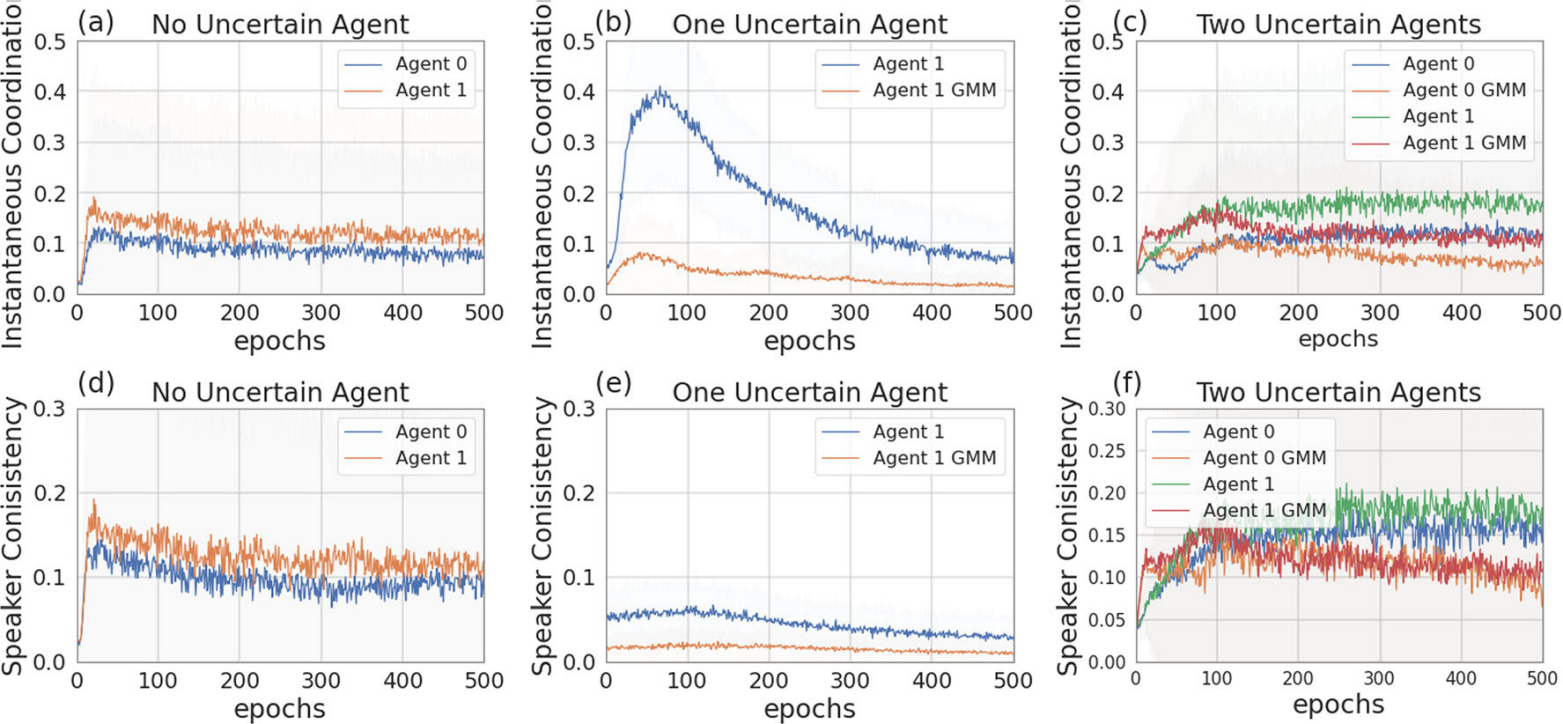
Instantaneous coordination and speaker consistency for the two-agent experiment for the three cases: full communication and no incentive uncertainty (left column), one uncertain agent (central column), two uncertain agents (right column). Agents are trained using REINFORCE

Figure 9 presents the four scenarios employed when two agents are uncertain, both without (images (a) and (b)) and with communication (images (b) and (c)) among the learning agents, and both without (images (a) and (c)) and with (images (b) and (d)) GMM model for uncertainty. The returns during training for this scenario, when gents are trained using the REINFORCE algorithm (and $\sigma =0.5$) are shown in Fig. 10, and Fig. 11 reports the probability of cooperating of the two agents during training. From the plots, we can observe again that adding uncertainty diminishes the capacity of the two agents to differentiate between scenarios where cooperation or defection is rational. This happens especially when $f \in \{1.5, 2.5\}$: at $f=2$, the nature of the game shifts from competitive ($f < 2$) to cooperative ($f > 2$). Therefore, in the surroundings of this turning point, the presence of uncertainty makes it harder for the agents to understand which kind of game they are playing. The same results have been found by employing the DQN algorithm (Fig. 18). We introduce a bidirectional communications step in this scenario: here both agents send and receive messages. From the two aforementioned figures, we can observe that there is no significant difference between the cooperation values the agents converge to at the end of the training when only the uncertainty modelling module is enabled with respect to when it is not, and no communication channel is used. (We tested this hypothesis with the Welch’s *t*-test using a threshold of 0.0001.) When only communication is enabled, but no model of uncertainty is used, the main effect of communication is to improve the cooperation probability of the agents in the mixed-motive game, while it has no impact on the others (see Table 7a). However, it is the combined effect of communication and uncertainty modelling that has the highest impact in aiding cooperation in the mixed-motive scenario. Indeed, when $f=1.5$ those move the equilibria of the game cooperation with average probability 0.15, to cooperation with an average probability of 0.67 (see Table 7b). As Fig. 12 shows, speaker consistency signals that in this setting both agents send reliable messages (Fig. 12f). The instantaneous coordination shows a nonzero amount of information transfer as well (Fig. 12c). We also observe how the agents cooperate with higher probability in the mixed-motive scenario ($f=1.5$), when uncertainty and communication are present (the average cooperation probability is 0.67, Table 7b), with respect to the scenario with full observability and communication (average cooperation probability 0.32, Table 3). This effect is worth investigating further and will be part of our future work.

**Table 1:**
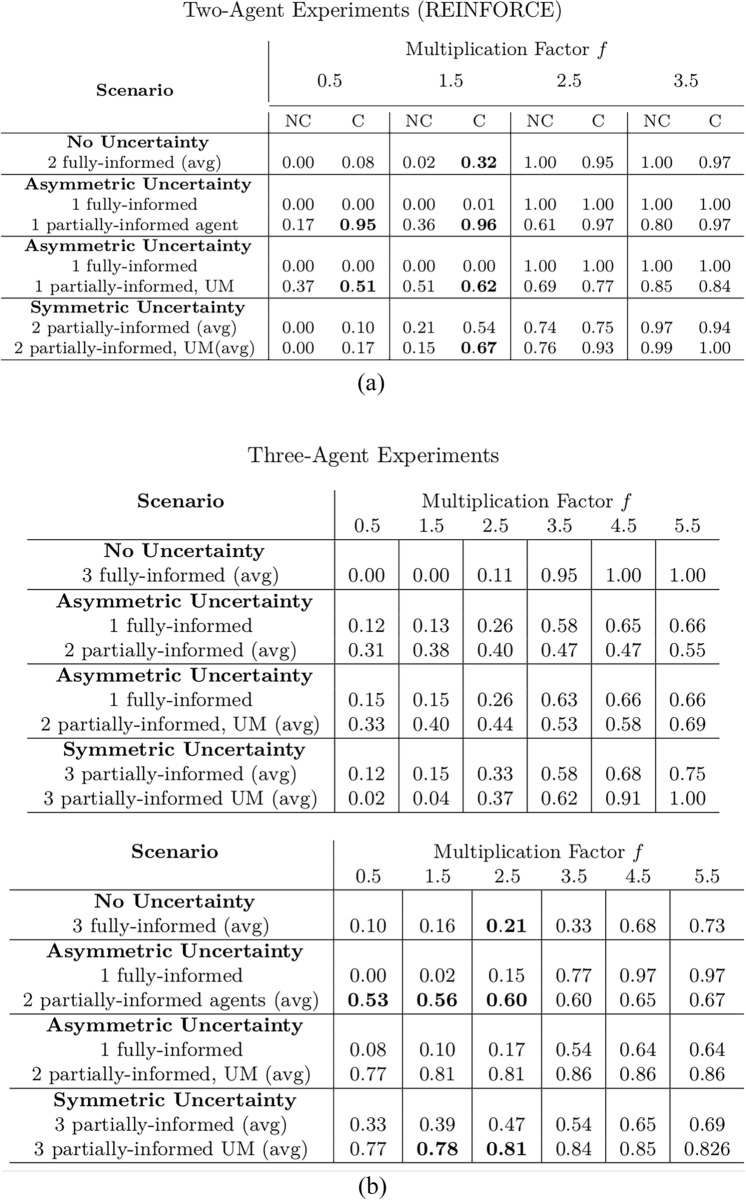
Cooperation probabilities for the two-agent (1a) and three-agent (1b) scenarios, obtained with the REINFORCE algorithm. We present the non-communication (NC) and communication (C) cases, with uncertainty modelling (UM) and without. When more agents have the same characteristics, the average measure is reported. The bold values highlight the principal outcomes (where the difference between the outcomes with and without communication is statistically significant)

**Table 2:**
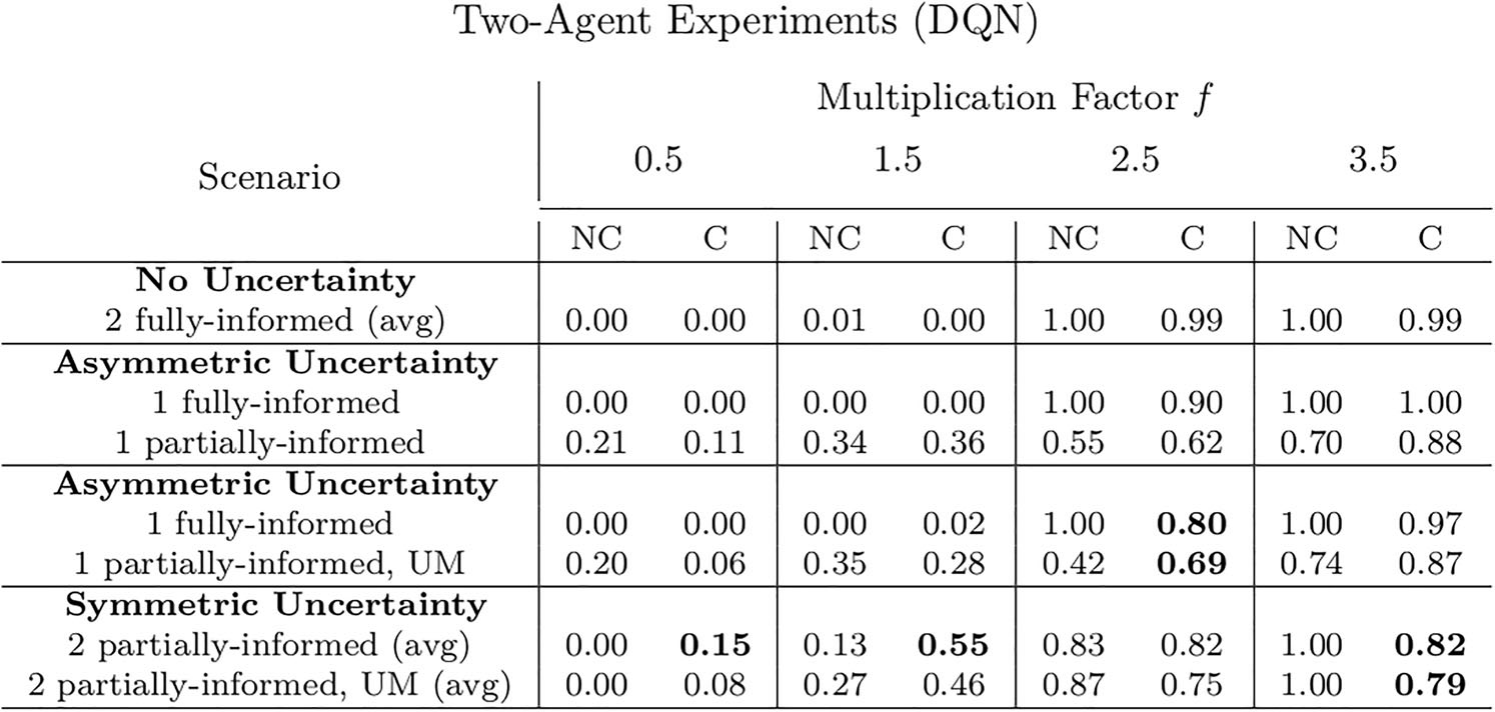
Cooperation probabilities for the two-agent scenarios, obtained with the DQN algorithm. We present the non-communication (NC) and communication (C) cases, with uncertainty modelling (UM) and without. When more agents have the same characteristics, the average measure is reported. The bold values highlight the principal outcomes (where the difference between the outcomes with and without communication is statistically significant)

### Three-player games

For the three-player scenario, we ran experiments with the following set of multiplication factors: $F = \{ 0.5, 1.5, 2.5, 3.5, 4.5, 5.5 \}$. These values are chosen following the criterion defined at the beginning of Sect. 5, namely, so that in the scenarios without incentive uncertainty the agents converge to the dominant strategies. In this setting, the dominant strategy for rational agents is to defect when $f \in \{0.5, 1.5, 2.5\}$, and cooperate when $f \in \{3.5, 4.5, 5.5\}$. Here, we refer to $f \in \{3.5, 4.5, 5.5\}$ as cooperative games, to $f \in \{1.5, 2.5\}$ as mixed-motive ones and to $f=0.5$ as the competitive one. We perform experiments for three different training scenarios: 1) all agents receive perfect observations of the multiplication factor *f*; 2) two of the three agents receive uncertain observations of *f*; and 3) all three agents receive uncertain observations of *f*.

Like in the previous section, we focus on the main findings and we collect complete data about the data and analysis in Appendix A.

**Fig. 13:**
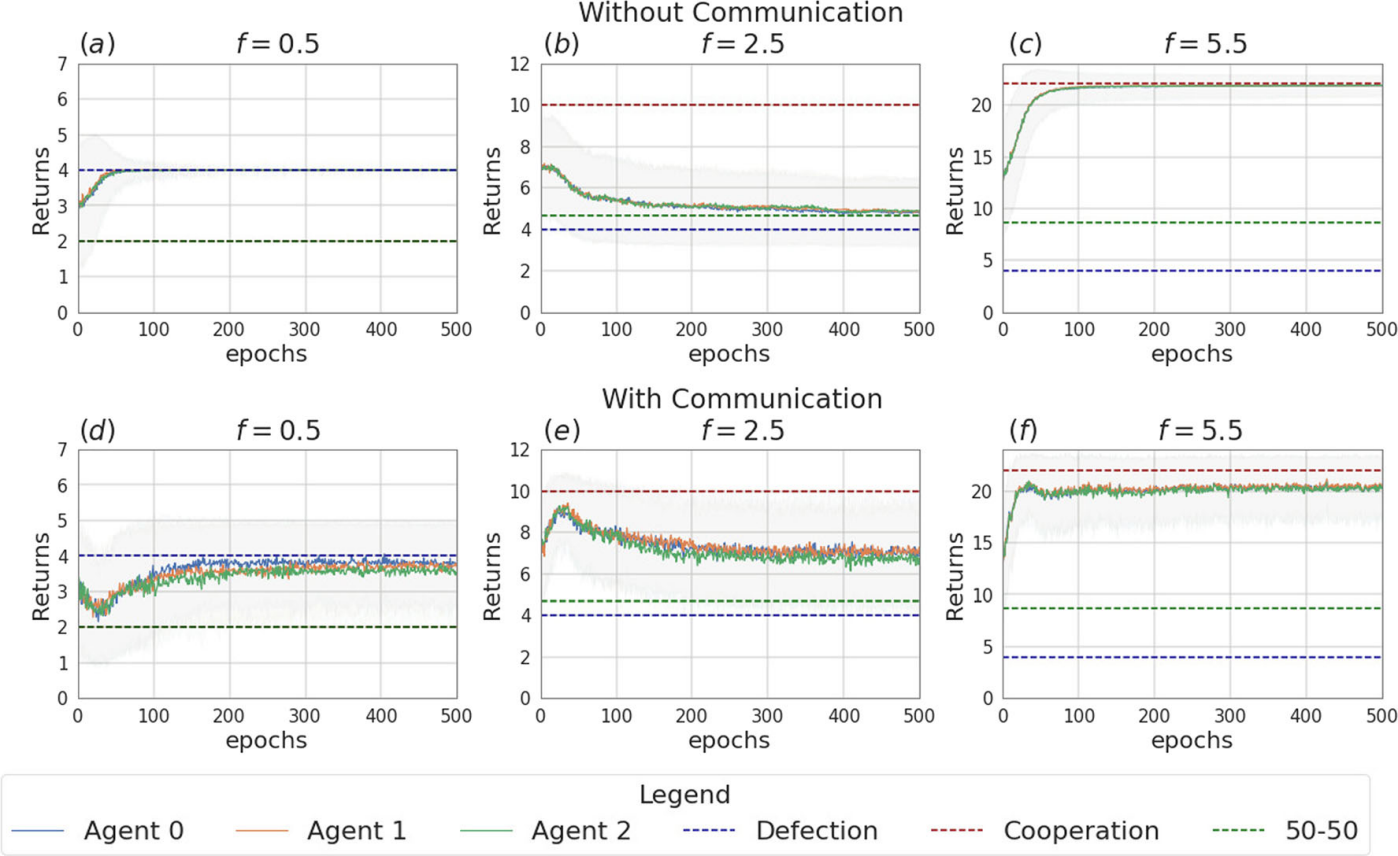
Returns during training for the setting with three agents and without uncertainty, in the scenarios without communication (top row) and with communication (bottom row). The curves are averages over 80 runs. The horizontal dashed lines represent the returns the agents would obtain if they always cooperate (dashed red line), always defect (dashed blue line), cooperate or defect with probability 0.5 (dashed green line). Agents are trained using REINFORCE

**Fig. 14:**
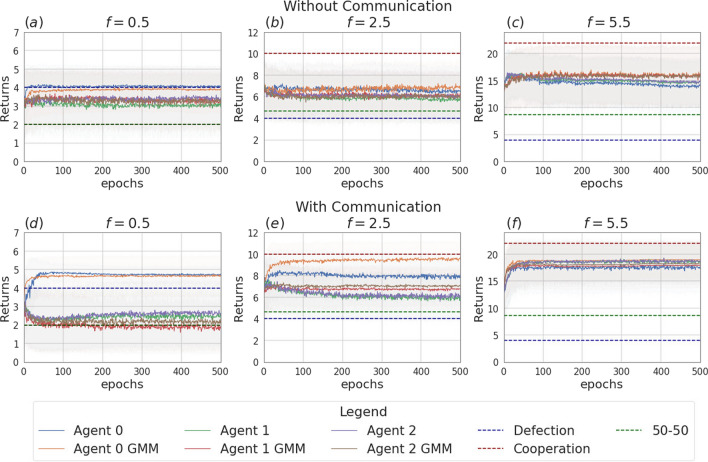
Returns during training for the setting with three agents and two uncertain agents (agent 1 and agent 2), in the scenarios without communication (top row) and with communication (bottom row). The curves are averages over 80 runs. The horizontal dashed lines represent the returns the agents would obtain if they always cooperate (dashed red line), always defect (dashed blue line), cooperate or defect with probability 0.5 (dashed green line). Agents are trained using REINFORCE

**Fig. 15:**
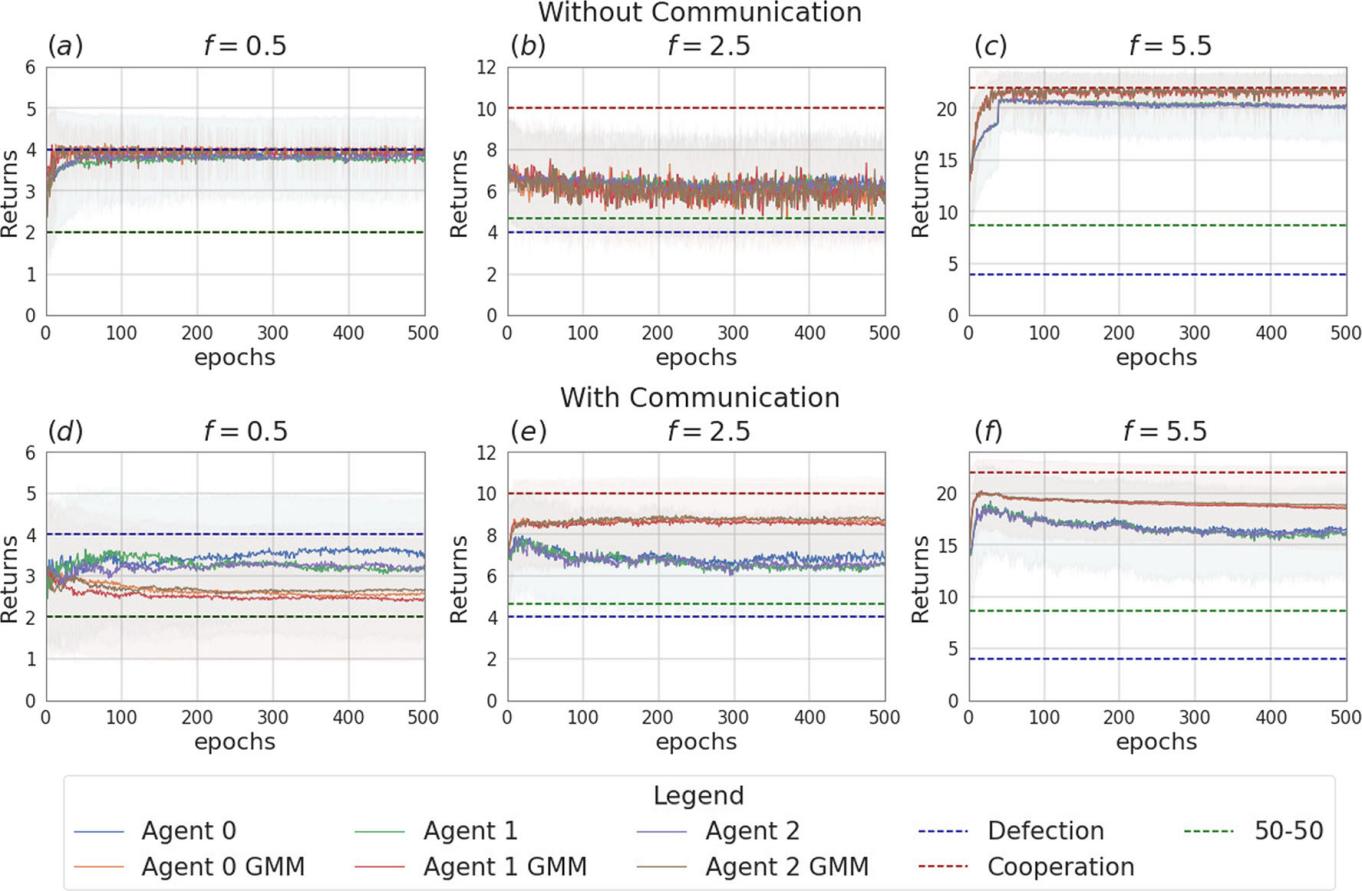
Returns during training for the setting with three uncertain agents, in the scenarios without communication (top row) and with communication (bottom row). The curves are averages over 80 runs. The horizontal dashed lines represent the returns the agents would obtain if they always cooperate (dashed red line), always defect (dashed blue line), cooperate or defect with probability 0.5 (dashed green line). Agents are trained using REINFORCE

**Fig. 16:**
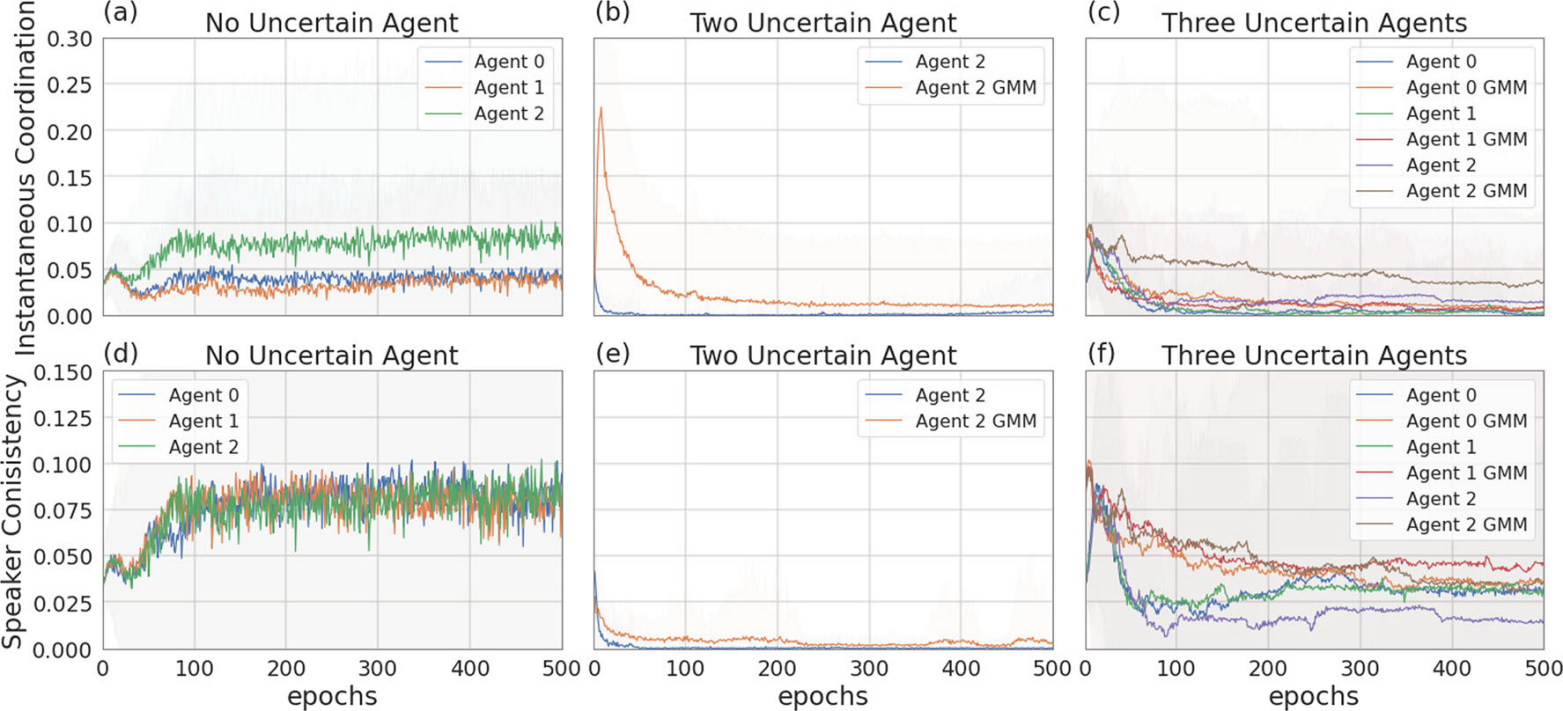
Instantaneous coordination and speaker consistency for the three-agent experiment for the three cases: full communication and no incentive uncertainty (left column), two uncertain agents (central column), three uncertain agents (right column). Agents are trained using REINFORCE

The experiments conducted in the scenario with three agents and no incentive uncertainty, yield outcomes similar to those observed in the two-agent case. In the scenario without communication, agents converge towards cooperation in cooperative games and to defection in competitive scenarios (see Table 9). The returns for a subset of the employed multiplication factors ($f \in \{0.5, 2.5, 5.5\}$) are shown in Fig. 13. Figure 16 illustrates the instantaneous coordination and the speaker consistency measures computed at each learning iteration for the three different experiments and will be used to discuss observations available through the returns plots of the three experiments (Figs. 13, 14, 15).

When communication is allowed, at each iteration of the training all agents generate a message. These messages are then concatenated and used as input for the action policies of all three agents. We observe that enabling message exchange among agents increases the average cooperation probability in the mixed-motive settings (as in the two-agent scenario) and in the competitive one, but sensitively decreases it in the cooperative games (see Table 9). We argue that the reason for this is that, due to the lack of necessity for message exchange in this scenario, the message input results in constituting just a noisy input for the actions policies’ networks, which hinders the network’s ability to disentangle the useful information and perform correct predictions.

Figure 14 shows the returns of the agents in the scenario in which two of the three agents receive uncertain observation (agent 1 and agent 2, with $\sigma = 2$). We again observe that, in the setting without communication, the uncertain agents struggle to disentangle when it is convenient to cooperate from when it is convenient to defect. This uncertainty affects the certain agent as well (agent 0), which can no longer perfectly converge to the dominant strategies, especially in the cooperative games (see Table 10a and b). The communication step is introduced in this setting such that only the certain agent sends a message, which is received by the two uncertain agents. As we observed in the previous section, the introduction of message exchange, when the uncertainty modelling module is disabled, carries a positive outcome on the returns collected by the certain agent in all the six games. Regarding the uncertain agents, the communicative input has a negative impact on the collected returns in the competitive game and mixed-motive games and a positive one on the returns collected in the cooperative games: the uncertain agents end up cooperating more in all six games, where the effect of communication is significantly more impactful when $f \in \{0.5, 4.5, 5.5\}$. The curves showing the instantaneous coordination and speaker consistency in this scenario (Fig. 16b, e) indicate that the speaker consistency of agent 0 is low, meaning that the content of the messages sent by the agent is not consistent over different instances. The fact that instantaneous coordination of agents 1 and 2 becomes very low during training (Fig. 16e) means that the uncertain agents learn to not trust the messages of the certain agent. We argue that the main effect of communication in this case is to represent a noisy input for the network of the action’s policies: with respect to what we observed in the two-agent scenarios, we believe deception is most likely not happening in this case; however, the reason why this noisy input constitutes a positive bias in the cooperation probability for the uncertain agents is still unclear. This result holds both when the uncertainty is and is not modelled by the GMM.

The main outcomes from the experiments in the scenario where all three agents are uncertain ($\sigma = 1$) are consistent with the ones obtained in the scenario with two learning agents. The returns of the agents for $f \in \{0.5, 2.5, 5.5\}$ are shown in Fig. 15. In this setting, the effect of uncertainty (i.e. decreasing the ability of agents to distinguish the situations in which it is rational to cooperate or defect) is evident, especially for $f \in \{1.5, 2.5, 3.5\}$, a set which contains all the games with multiplication factor near to $f=3$, which is the turning point from which the equilibrium moves from competitive $f<3$ to cooperative $f>3$. In the surroundings of this value, the presence of uncertainty can make it so that the agents might get observations of a certain nature (e.g. a multiplication factor corresponding to a cooperative game) that differ from the true game being played (e.g. a mixed-motive one). Adding the uncertainty modelling module in this scenario enables the agents to partially regain information and therefore shift the learned behaviour nearer to the rational strategy in all the six games when communication is not enabled (see Table 7a and 7b). The difference in the addition of the GMM module though is statistically significant only when $f \in \{0.5, 5.5\}$, where the addition of the uncertainty modelling allows the agents to manage to get significantly closer to the rational strategy. In all the other games, the convergence is not achieved.

We introduced a communication step in this scenario: in this case, before acting, every agent sends a message. When no uncertainty modelling module is used, the main effect of adding the communication channel to this scenario is to improve the cooperation among the agents when $f \in \{0.5, 1.5, 2.5\}$ (see Table 10a). However, it is again the combined effect of the communication channel and the uncertainty modelling module that aids cooperation the most in these three games (see Table 10b): the average cooperation value moves from 0.02 to 0.77 when $f=0.5$, 0.04 to 0.78 when $f=1.5$ and 0.37 to 0.81 when $f=2.5$. Moreover, we observe again that the agents cooperate with a significantly higher probability in the mixed-motive scenarios, when uncertainty and communication are present, with respect to the scenario with no uncertainty and no communication. As already mentioned, the deepening of the reasons for this outcome will be part of our future work. As Fig. 16 shows, consistently with the two-agent scenario, the speaker consistency measure shows that agents send reliable messages (Fig. 16c), and there is a nonzero amount of information transfer: here the communication channel is being employed by the agents for all the duration of the learning (Fig. 16f).

## Conclusion

In this paper, we investigated the effects of emergent communication on independent reinforcement learning agents trained on a spectrum of environments with different incentive alignments and in the face of environmental uncertainty. Our findings indicate that adding uncertainty over one agent’s observations worsens its performance, as the agent cannot effectively disentangle the situations where cooperation would be favourable from those in which it is not. We showed that in cases of asymmetric uncertainty—where only a subset of agents receives uncertain observations—the communication channel can be employed by the agents that receive observations without uncertainty to deceive the uncertain ones. As a result, certain agents experience higher rewards while the uncertain ones receive lower rewards. Furthermore, we observed how learning agents with equal levels of uncertainty can leverage the combined effects of communication and uncertainty modelling to enhance group cooperation. This enables them to overcome the dominated equilibria of mixed-motive scenarios, ultimately improving the social welfare of the group.

We believe that the employment of emergent communication in mixed cooperative–competitive multi-agent systems can prove to be an effective strategy for the development of reliable artificial agents, and therefore, for cooperative AI. As future work, we plan to: provide theoretical underpinnings to the observed combined effect of uncertainty and communication in improving levels of cooperation in mixed-motives interactions; investigate the learning dynamics studied in this paper in larger settings; and investigate the effect of social structures (e.g. reputation and norms [33]) and different communication frameworks (e.g. graph neural networks [30]).

## Figures and Tables

**Table 5:**
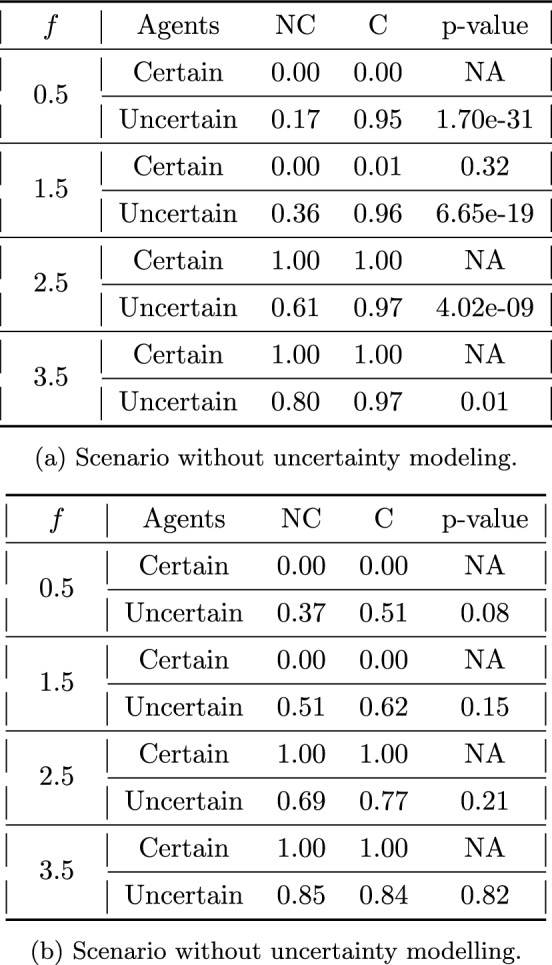
Cooperation probabilities computed for each game in the scenario with two agents where one receives uncertain observations, trained using the REINFORCE algorithm. We reported the *p* values resulting from Welch’s *t*-tests among the scenarios with and without communication

**Table 6:**
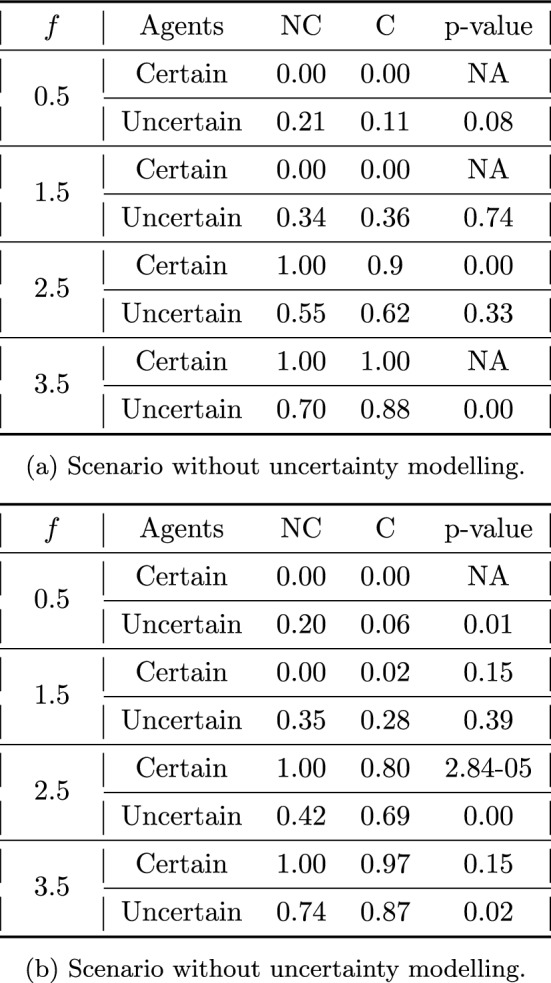
Cooperation probabilities computed for each game the scenario with two agents where one receives uncertain observations, trained using the DQN algorithm. We reported the *p* values resulting from Welch’s *t*-tests among the scenarios with and without communication

**Table 7:**
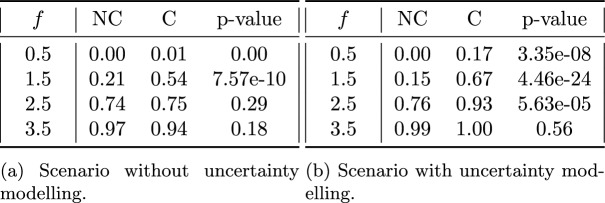
Cooperation probabilities computed for each game in the scenario with two uncertain agents, trained using the REINFORCE algorithm. We reported the *p* values resulting from Welch’s *t*-tests among the scenarios with and without communication

**Table 8:**
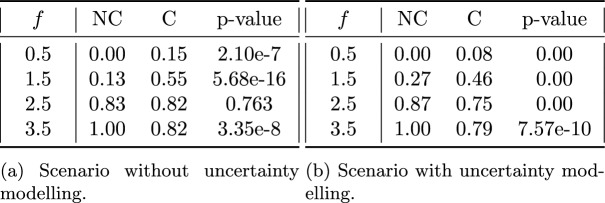
Cooperation probabilities computed for each game in the scenario with two uncertain agents, trained using the DQN algorithm. We reported the *p* values resulting from Welch’s *t*-tests among the scenarios with and without communication

**Table 9:**
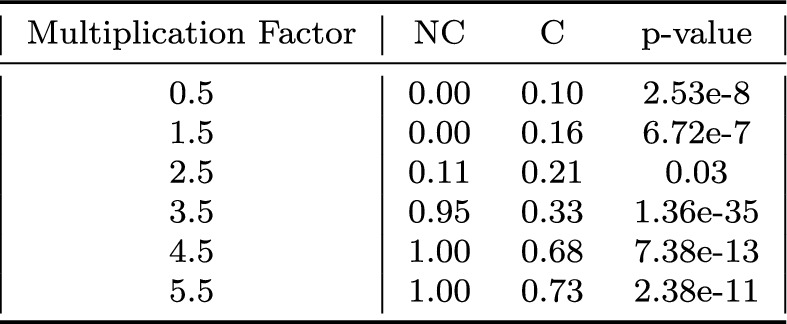
Cooperation probabilities computed for each game in the scenario with three agents and no incentive uncertainty, trained using the REINFORCE algorithm. We reported the *p* values resulting from Welch’s *t*-tests among the scenarios with and without communication

**Table 10:**
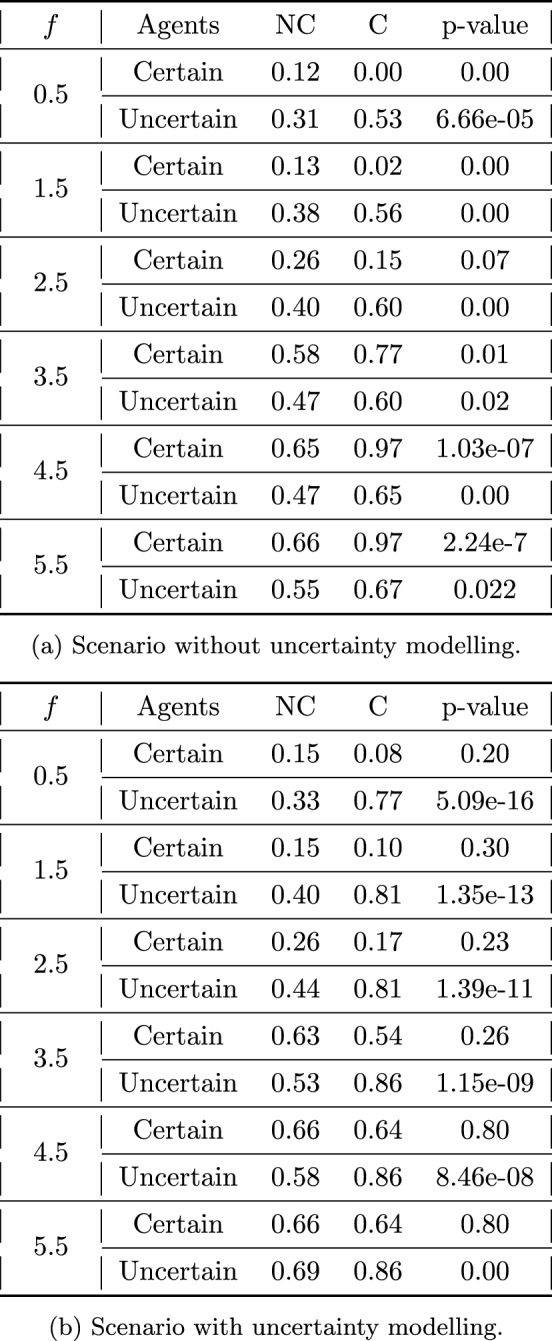
Cooperation probabilities computed for each game in the scenario with three agents where two receive uncertain observations, trained using the REINFORCE algorithm. We reported the *p* values resulting from Welch’s *t*-tests among the scenarios with and without communication

**Table 11:**
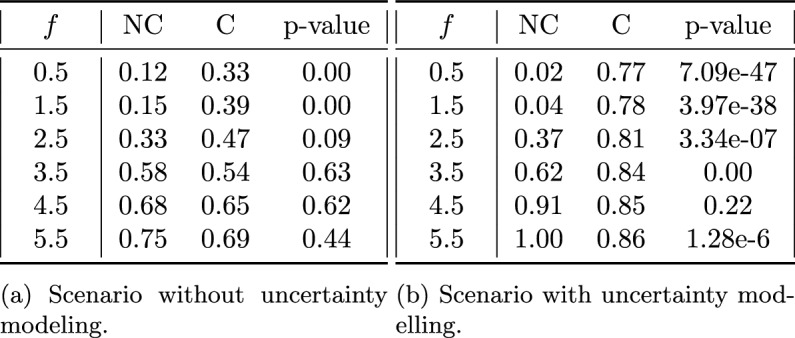
Cooperation probabilities computed for each game in the scenario with three uncertain agents, trained using the REINFORCE algorithm. We reported the *p* values resulting from Welch’s *t*-tests among the scenarios with and without communication

**Fig. 17:**
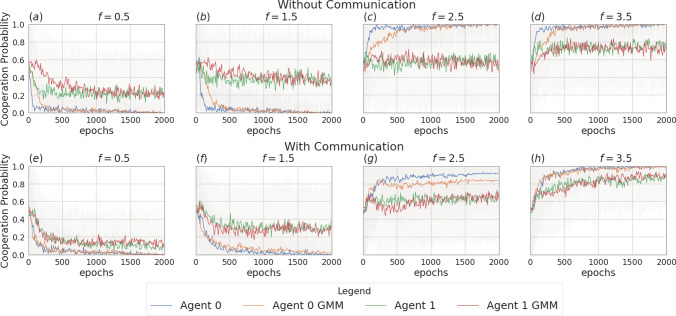
Probability of cooperating during training for agents trained using DQN, in the scenario with one uncertain agent (agent 1, with $\sigma =2$), in the non-communication case (upper row) versus communication case (lower row)

**Fig. 18:**
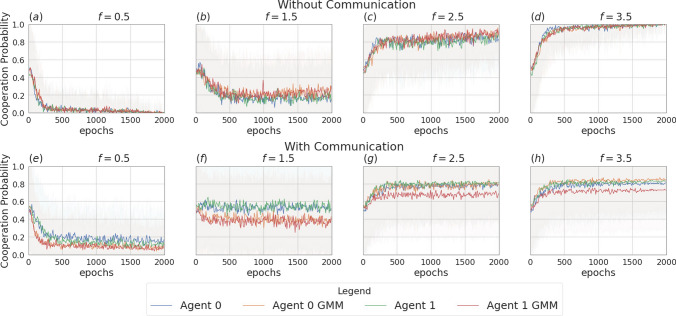
Probability of cooperating during training, in the scenario with two uncertain agents ($\sigma =0.5$), trained using DQN, in the non-communication case (upper row) versus communication case (lower row)

**Fig. 19:**
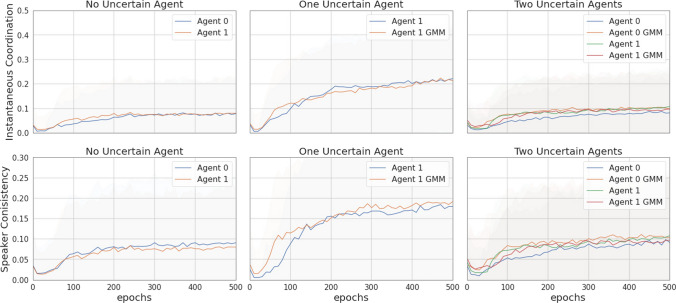
Instantaneous coordination and speaker consistency for the two-agent experiment trained with DQN, for the three cases: full communication and no incentive uncertainty (left column), one uncertain agent (central column), two uncertain agents (right column)

**Fig. 20:**
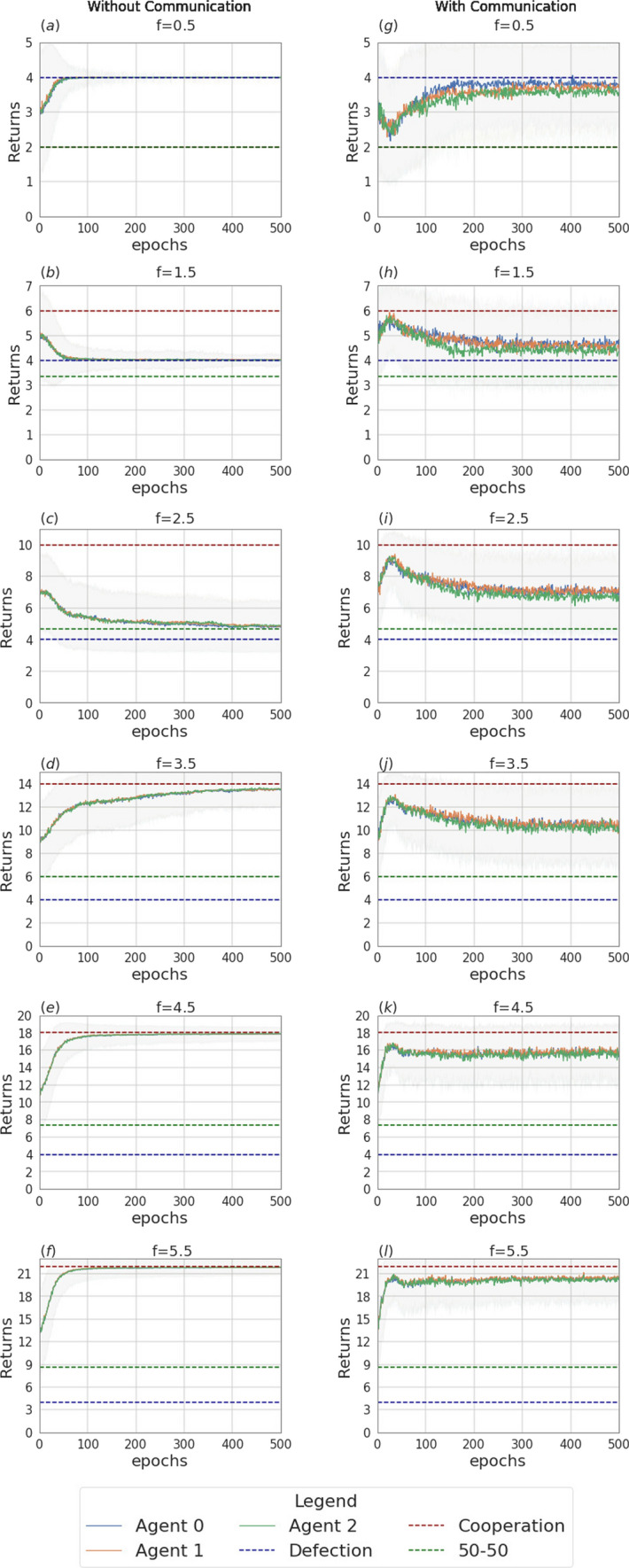
Returns during training, in the three-agent scenario with no incentive uncertainty in the non-communication case (left column) versus communication case (right column). The horizontal lines represent the returns agents would obtain if they always cooperate (dashed red line), always defect (dashed blue line), cooperate or defect with probability 0.5 (dashed green line)

**Fig. 21:**
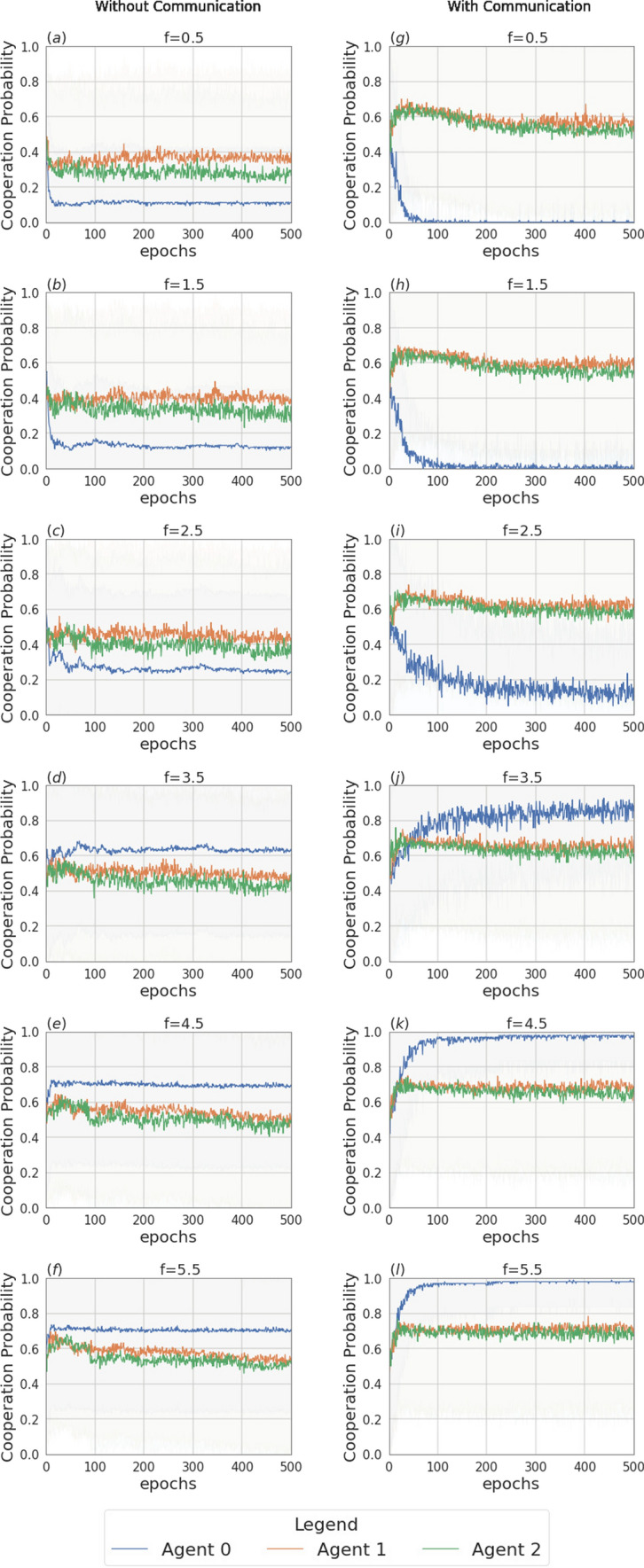
Cooperation probability during training, in the three-agent scenario with no incentive uncertainty in the non-communication case (left column) versus communication case (right column). The horizontal lines represent the returns agents would obtain if they always cooperate (dashed red line), always defect (dashed blue line), cooperate or defect with probability 0.5 (dashed green line)

**Fig. 22:**
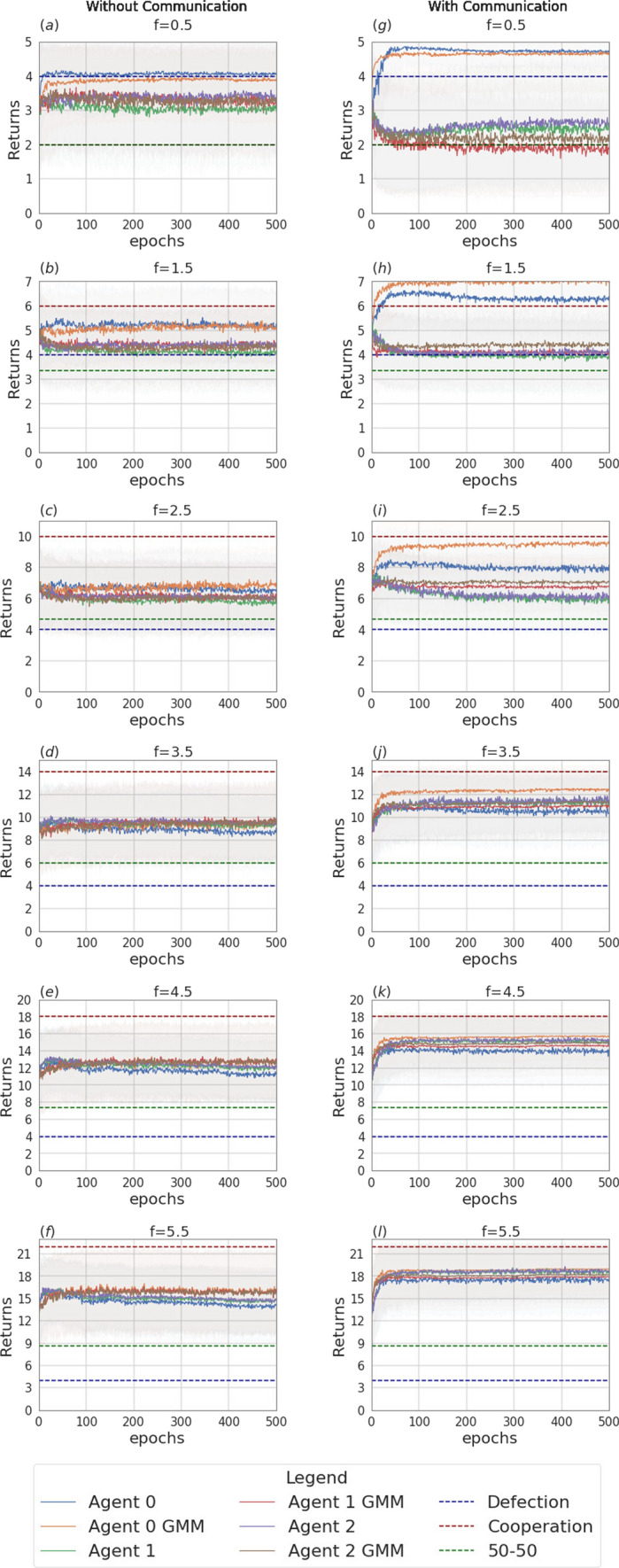
Returns during training, in the three-agent scenario where two agents receive uncertain observations (agent 1 and agent 2, with $\sigma =2$), in the non-communication case (left column) versus communication case (right column). The horizontal lines represent the returns agents would obtain if they always cooperate (dashed red line), always defect (dashed blue line), cooperate or defect with probability 0.5 (dashed green line)

**Fig. 23:**
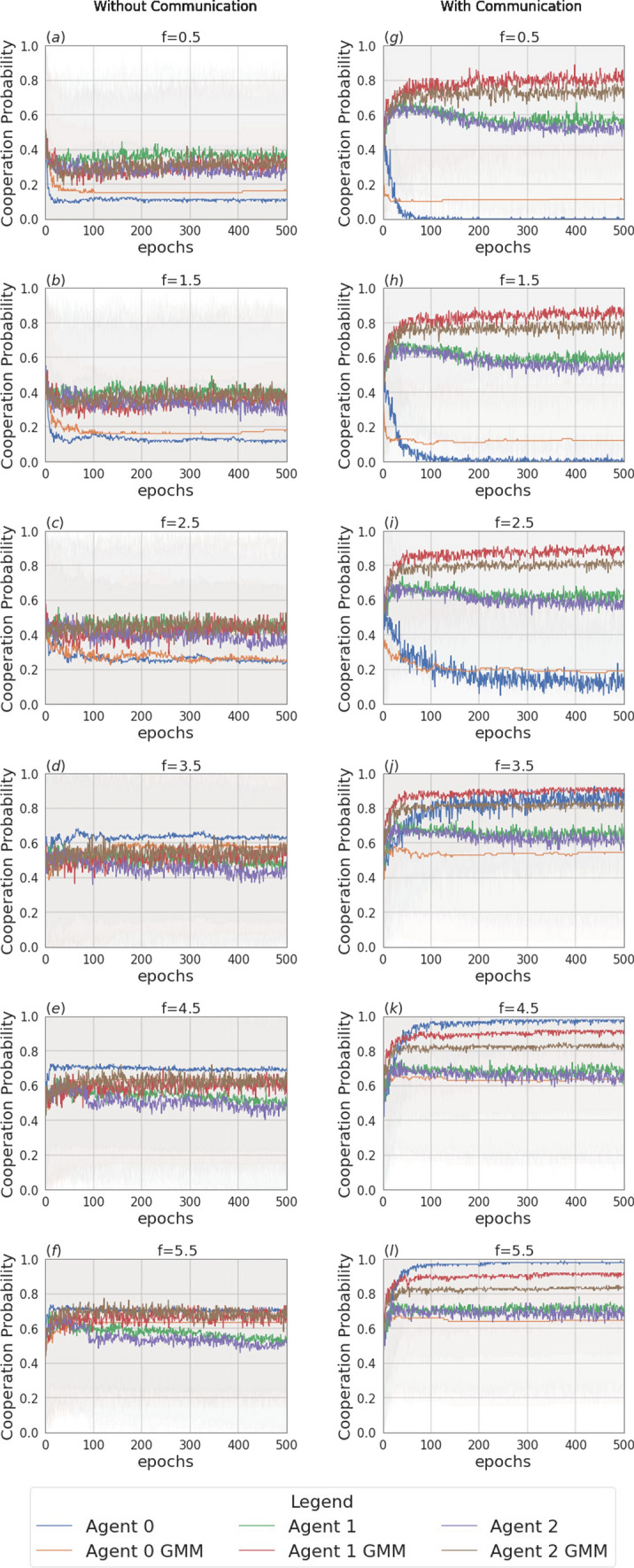
Cooperation probabilities during training, in the three-agent scenario where two agents receive uncertain observations (agent 1 and agent 2, with $\sigma =2$), in the non-communication case (left column) versus communication case (right column)

**Fig. 24:**
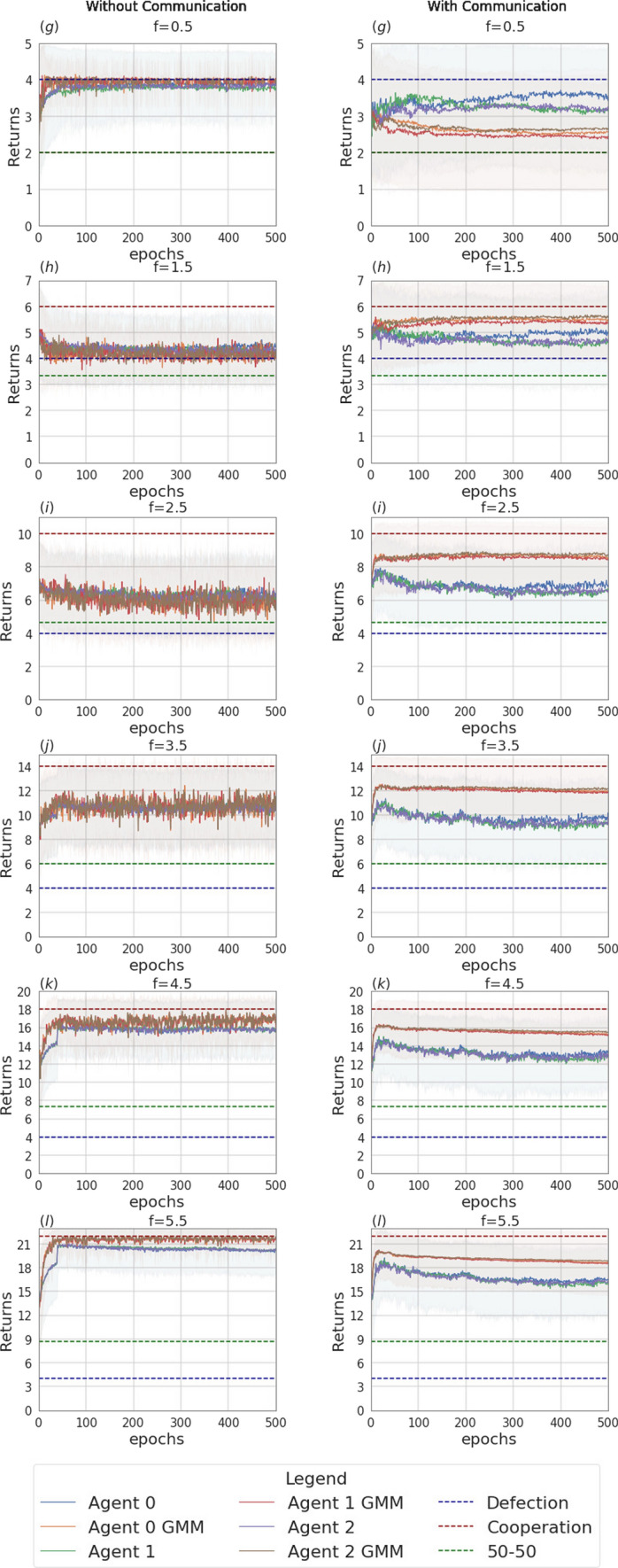
Returns during training, in the three-agent scenario with all agents uncertain ($\sigma =1$), in the non-communication case (left column) versus communication case (right column). The horizontal lines represent the returns agents would obtain if they always cooperate (dashed red line), always defect (dashed blue line), cooperate or defect with probability 0.5 (dashed green line)

**Fig. 25:**
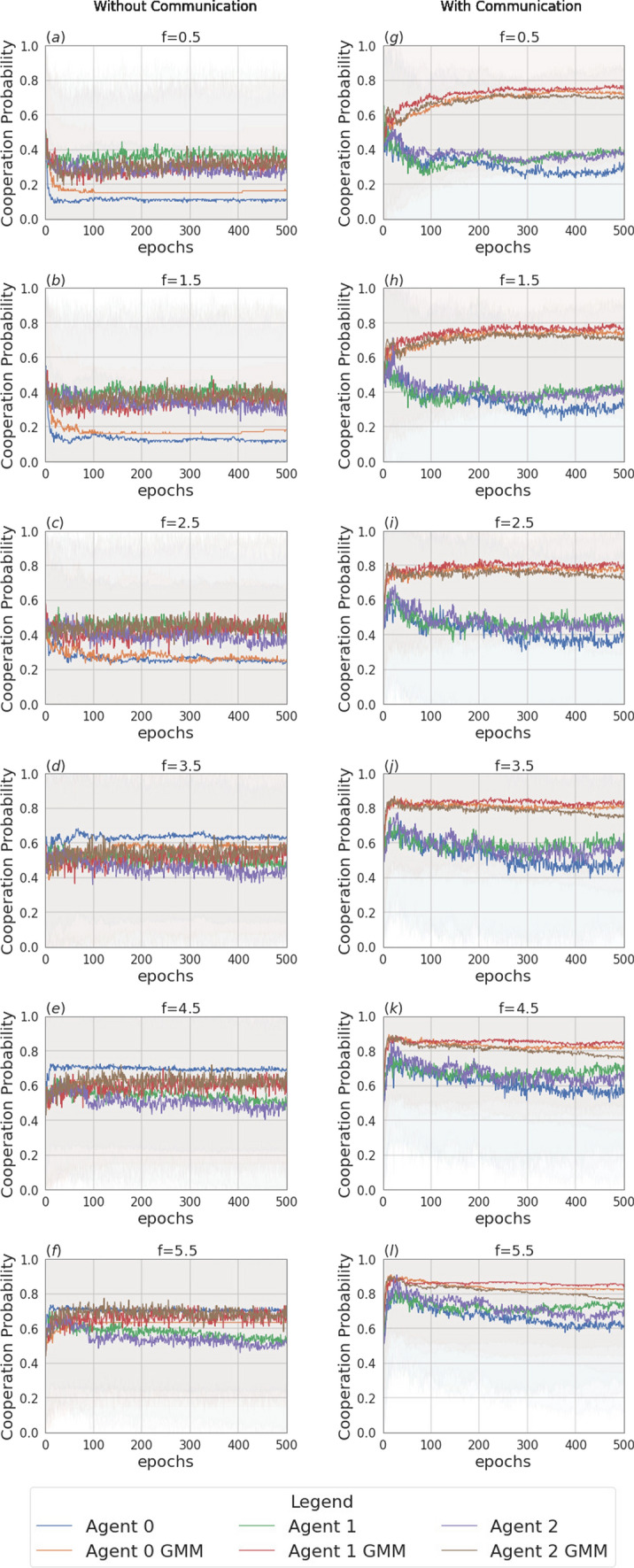
Cooperation values during training, in the three-agent scenario with all agents uncertain ($\sigma =1$), in the non-communication case (left column) versus communication case (right column)

## Data Availability

The source code of the environment and algorithms implemented and analysed in this study are available at https://github.com/nicoleorzan/marl-in-pgg.

## Appendix A: Statistical analysis

In this section of the appendix is enclosed additional information about the REINFORCE and DQN experiments with two and three players, with and without communication. In particular, we show the results in term of average cooperation values reached by the agents at the end of the training. Moreover, we perform Welch’s statistical testing against the scenarios with and without communication and report the results in terms of *p* value. When the *p* value is smaller than the threshold of 0.0001, the difference among the results is significant. NA is reported in the tables when no distribution can be extracted from the result, so no statistic can be computed.

### A.1 Two-agent experiments

Table 3 summarizes the probability of cooperation resulting from the experiments with no uncertainty when agents are trained using the REINFORCE algorithm and Table 4 for agents trained using DQN. Table 5a and b shows the results for the experiments with one uncertain agent (with uncertainty $\sigma =2$) when agents are trained using the REINFORCE algorithm, while Table 6a and b shows the results for agents trained using the DQN algorithm. Table 7a and b shows the results for the experiments with two uncertain agents (with uncertainty $\sigma =0.5$), trained with REINFORCE, and Table 8a and b the results for agents trained using DQN.

**Table 3:** Average cooperation probabilities computed for each game in the scenario with two agents and no incentive uncertainty trained using the REINFORCE algorithm. We reported the *p* values resulting from Welch’s *t*-tests among the scenarios with and without communication

Multiplication factor	NC	C	*p* value
0.5	0.00	0.08	0.00
1.5	0.02	0.32	4.70e$-$14
2.5	1.00	0.95	0.00
3.5	1.00	0.97	0.04

**Table 4:** Cooperation probabilities computed for each game in the scenario with two agents and no incentive uncertainty trained using the DQN algorithm. We reported the *p* values resulting from Welch’s *t*-tests among the scenarios with and without communication

Multiplication factor	NC	C	*p* value
0.5	0.00	0.00	NA
1.5	0.01	0.00	0.15
2.5	1.00	0.99	0.31
3.5	1.00	0.99	0.31

### A.2 Three-agent experiments

Table 9 presents the final average cooperation probabilities resulting from the experiments with no uncertainty on the observations, Table 10a and b the results for the experiments with two uncertain agents, and Table 11a and b the results for the experiments with three uncertain agents.

## Appendix B: Additional results

This section reports additional results relative to two-agent experiments performed using DQN and three-agent experiments performed using REINFORCE.

### B.1 Two agents

This subsection reports the figures relative to the two-agent experiments performed using DQN. In particular, Fig. 17 shows the average cooperation probability for the two-agent scenarios with one uncertain agent and $\sigma =2$, while Fig. 18 shows the results for the scenario with two uncertain agents with $\sigma =0.5$ (Fig. 12).

### B.2 Three agents

This subsection reports the figures relative to the three-agent experiments performed using the REINFORCE algorithm, for the cases with and without communication, when there is no uncertain agent (Figs. 20, 21), for the cases with two uncertainty agents, with uncertainty $\sigma =2$ (Figs. 22, 23) and for the cases with three uncertain agents, with uncertainty $\sigma =1$ (Figs. 24, 25).

## Appendix C: Hyperparameters

In this section, we report the hyperparameters employed for the two-agent and three-agent experiments, for the REINFORCE and DQN algorithms. In all the performed experiments, the number of training epochs has been set to 600 for the REINFORCE algorithm and to 2000 for DQN (Tables 12, 13, 14, 15).

**Table 12:** Selected hyperparameters for the two-agent scenarios without communication

Hyperparameter	Two certain agents	One uncertain agent	Two uncertain agents
Batch size	64	64	64
Hidden size $\pi _A$	8	8	16
N. hidden layers $\pi _A$	2	2	2
Learning rate $\pi _A$	0.017	0.017	0.004
Decay rate	0.999	0.999	0.999
Target net update freq (episodes)	30	30	30

**Table 13:** Selected hyperparameters for the two-agent scenarios with communication

Hyperparameter	Two certain agents	One uncertain agent	Two uncertain agents
Batch size	128	64	64
Hidden size $\pi _C$	64	64	64
Hidden size $\pi _A$	64	64	64
N. hidden layers $\pi _C$	1	1	1
N. hidden layers $\pi _A$	1	2	2
Learning rate $\pi _C$	0.075	0.04	0.04
Learning rate $\pi _A$	0.028	0.004	0.004
Listening loss weight	0.37	0.37	0.37
Signalling loss weight	0.52	0.52	0.52
Message size	9	8	8
Decay rate	0.999	0.999	0.999
Target net update freq (episodes)	30	30	30

**Table 14:** Selected hyperparameters for the three-agent scenarios without communication

Hyperparameter	Three certain agents	Two uncertain agent	Three uncertain agents
Batch size	64	64	64
Hidden size $\pi _A$	8	16	16
N. hidden layers $\pi _A$	2	2	2
Learning rate $\pi _A$	0.017	0.017	0.04
Decay rate	0.999	0.9Decay99	0.999
Target net update freq (episodes)	30	30	30

**Table 15:** Selected hyperparameters for the three-agent scenarios with communication

Hyperparameter	Three certain agents	Two uncertain agent	Three uncertain agents
Batch size	128	128	128
Hidden size $\pi _C$	8	64	8
Hidden size $\pi _A$	64	32	32
N. hidden layers $\pi _C$	1	2	2
N. hidden layers $\pi _A$	1	2	2
Learning rate $\pi _C$	0.075	0.005	0.02
Learning rate $\pi _A$	0.0532	0.059	0.021
Listening loss weight	0.68	0.77	0.32
Signalling loss weight	0.40	0.65	0.50
Message size	9	8	9
Decay rate	0.999	0.999	0.999
Target net update freq (episodes)	30	30	30

## Appendix D: Proof of Proposition 1

We present the proof of Proposition 1.

### Proof

*(i)* $$\boxed {0 \le f \le 1}$$.

- Let us show that $\varvec{s}_D$ is the only dominant strategy equilibrium. This means we need to show that any *s* other than $s_D$ is dominated by $s_D$.
  Let us pick a strategy profile $\varvec{s}\in S^n$, which differs from $\varvec{s}_D$, and in which *m* agents are being cooperative (with $0<m<n$); that is, for any such agent *k*, $s_k(C) >0$. Then, the utility of *k* in $\varvec{s}$ reads:
  D1 $$\begin{aligned} u_k(\varvec{s}_{C_k}, \varvec{s}_{-k}) = \frac{f}{n} \sum _{i=1}^{m} c_i + \frac{f}{n} c_k < \frac{f}{n} \sum _{i=1}^{m} c_i + c_k = u_k(\varvec{s}_{D_k} , \varvec{s}_{-k}) \end{aligned}$$
  where $u_k(\varvec{s}_{D_k}, \varvec{s}_{-k})$ denotes the strategy profile in which *k* deviates to $\varvec{s}_{D_k}$, since $\frac{f}{n} \le \frac{1}{n} < 1$. The statement follows since this is the case $\forall i \in N$, and $\varvec{s}_D$ is also the only dominant strategy equilibrium since we are considering only pure strategies.
- Let us show that $\varvec{s}_D$ is Pareto optimal. Reasoning by contradiction, assume that there exists a profile $\varvec{s}^{\prime }$ that Pareto dominates $\varvec{s}_D$.
  By definition of Pareto optimality, $\varvec{s}^{\prime } \ne \varvec{s}_D$. We want to show that $\forall \; i \in N$, $u_i(\varvec{s}^{\prime }) \ge u_i(\varvec{s}_D)$ and $\exists i \in N \; s.t. \; u_i(\varvec{s}^{\prime }) > u_i(\varvec{s}_D)$ leads to a contradiction. Without loss of generality, assume that the first *m* agents of profile $\varvec{s}^{\prime }$ are cooperative, with $m>0$.^4^ Then:
  D2 $$\begin{aligned} u_k(\varvec{s}^{\prime }) = {\left\{ \begin{array}{ll} \frac{f}{n} \sum\limits_{i=1}^{m} c_i &  {\text {if }} k\le m ~\text {(cooperative)}\\ \frac{f}{n} \sum\limits_{i=1}^{m} c_i + c_k &  {\text {if }} k>m ~\text {(competitive)} \end{array}\right. } \end{aligned}$$
  Thus, we have:
  D3 $$\begin{aligned} \sum _{k=0}^n u_k(\varvec{s}^{\prime }) = f \sum _{i=1}^m c_i > \sum _{i=1}^m c_i = \sum _{k=0}^n u_k(\varvec{s}_D) \end{aligned}$$
  which is a contradiction.

*(ii)* $$\boxed {1 < f \le n}$$

- Let us show that $\varvec{s}_D$ is a weakly dominant strategy equilibrium. We need to show that $u_i(\varvec{s}_{D_i}, \varvec{s}_{-i}) \ge u_i(\varvec{s}^{\prime }_i, \varvec{s}_{-i}) \; \forall i \in N$.
  Consider a system of *n* agents with *m* cooperative agents and $m>0$. Let $\varvec{s}$ be the strategy profile of that system. The utility of a cooperative or a defective agent *k* is given by Eq. D2. Following the same argument of *(*i), i.e. since $\frac{f}{n} \le 1$, $u_k(\varvec{s}_{D_k}, \varvec{s}_{-k}) \ge u_k(\varvec{s}_{C_k}, \varvec{s}_{-k}), \; \forall i \in N$, $\varvec{s}_D$ is weakly dominant over $\varvec{s}_C$, which is the only other pure strategy.
- Let us show that $\varvec{s}_C$ is Pareto optimal. Reasoning by contradiction, assume that there is a profile $\varvec{s}^{\prime }$ that Pareto dominates $\varvec{s}_C$. By definition of Pareto optimality, $\varvec{s}^{\prime } \ne \varvec{s}_C$, and $\forall \; i \in N$, $u_i(\varvec{s}') \ge u_i(\varvec{s}_C)$ and $\exists \; i \in N$ such that $u_i(\varvec{s}') > u_i(\varvec{s}_C)$. Consider a system of *n* agents with *m* cooperative agents, and $m < n$. For any cooperative agent, *k* should hold:
  D4 $$\begin{aligned} u_k(\varvec{s}') = \frac{f}{n} \sum _{i=1}^{m} c_i \ge \frac{f}{n} \sum _{i=1}^{n} c_i = u_k(\varvec{s}_C) \end{aligned}$$
  Since $m < n$ and $f/n < 1$, the inequality does not hold, which is a contradiction.

*(iii)* $$\boxed {f > n}$$

- Let us show that $\varvec{s}_C$ is the only dominant strategy equilibria. We need to show that $u_i(\varvec{s}_{C_i}, \varvec{s}_{-i}) \ge u_i(\varvec{s}^{\prime }_i, \varvec{s}_{-i}) \; \forall i \in N$, and exist $\varvec{s}^{*}_{-i}$ such that $u_i(\varvec{s}_{C_i}, \varvec{s}^{*}_{-i}) \ge u_i(\varvec{s}^{\prime }_i, \varvec{s}^{*}_{-i})$.
  Following the same argument of (*i*), let us pick a system of *n* agents with *m* cooperative agents, and $n-m \ne 0$. Let $\varvec{s}$ be the strategy profile of that system. The utility of a cooperative or a defective agent *k* in the system is given by Eq. D2. Since $\frac{f}{n} > 1$, $u_k(\varvec{s}_{C_k}, \varvec{s}_{-k}) > u_k(\varvec{s}_{D_k}, \varvec{s}_{-k}), \; \forall i \in N$, $\varvec{s}_C$ is dominant over $\varvec{s}_D$. Note that $\varvec{s}_C$ is also the only dominant strategy equilibrium since we are considering only pure strategies.
- Let us show that $\varvec{s}_C$ is Pareto optimal. Following the argument of $\textit{(i)}$ we reason by contradiction, assuming that there is a profile $\varvec{s}^{\prime }$ that Pareto dominates $\varvec{s}_C$.
  By definition of Pareto optimality, $\varvec{s}^{\prime } \ne \varvec{s}_C$. The system of utilities for $u_i(\varvec{s}') \ge u_i(\varvec{s}_C) \; \forall \; i \in N$ reads:
  D5 $$  \left\{ {\begin{array}{*{20}l}    {\frac{f}{n}\sum\limits_{{i = 1}}^{m} {c_{i} }  \ge \frac{f}{n}\sum\limits_{{i = 1}}^{n} {c_{i} } } \hfill  \\     \ldots  \hfill  \\    {\frac{f}{n}\sum\limits_{{i = 1}}^{m} {c_{i} }  \ge \frac{f}{n}\sum\limits_{{i = 1}}^{n} {c_{i} } } \hfill  \\    {\frac{f}{n}\sum\limits_{{i = 1}}^{m} {c_{i}  + c_{{m + 1}} }  \ge \frac{f}{n}\sum\limits_{{i = 1}}^{n} {c_{i} } } \hfill  \\     \ldots  \hfill  \\    {\frac{f}{n}\sum\limits_{{i = 1}}^{m} {c_{i}  + c_{n} }  \ge \frac{f}{n}\sum\limits_{{i = 1}}^{n} {c_{i} } } \hfill  \\   \end{array} } \right.  $$
  With at least one inequality being strict. Summing up the right- and the left-hand sides of the system of inequalities, we get:
  D6 $$\begin{aligned} \sum _{i=m+1}^n c_i \ge f \sum _{i=m+1}^{n} c_i \end{aligned}$$
  which leads to a contradiction for $f>n$.

$$\square $$

## Footnotes

1 By “non-situated”, we are referring to a scenario where agents are limited to taking purely communicative actions that have no impact on the state of the environment.

2 GMMs are a flexible soft-clustering mechanism that allows the modelling of many possible distributions of data. We implement the GMM module using Scikit-Learn’s Gaussian mixture class [50].

3 See https://github.com/nicoleorzan/marl-in-pgg.
